# Frequency of use and sonority sequencing in first- and second-language consonant cluster perception: facilitation is language-specific

**DOI:** 10.3389/fpsyg.2025.1483046

**Published:** 2025-08-18

**Authors:** Sophia Wulfert, Peter Auer, Adriana Hanulíková

**Affiliations:** ^1^Department of German Studies, University of Freiburg, Freiburg, Germany; ^2^English and American Studies, TU Braunschweig, Braunschweig, Germany; ^3^Freiburg Institute for Advanced Studies, University of Freiburg, Freiburg, Germany; ^4^Institut für Deutsch als Fremdsprachenphilologie, Heidelberg University, Heidelberg, Germany

**Keywords:** consonant cluster, frequency of use, phonotactics, Sonority Sequencing Principle, sublexical speech processing, speech perception, L2 perception

## Abstract

**Introduction:**

Expectations derived from knowledge about the likelihood of different phoneme sequences are an effective cognitive mechanism to make the listening process more efficient. In addition to language-specific distributions, universal principles of well-formedness may play a role, especially in second language listening, where the listeners are less familiar with the target language. In our study, we compared two listener groups to investigate the relative influences of consonant cluster frequency and consonant sequencing in accordance with the Sonority Sequencing Principle on the perception of initial consonant clusters in German.

**Methods:**

In Experiment 1, first-language (L1) German listeners identified noise-embedded nonce words with initial consonant clusters. In Experiment 2, Australian learners of German completed the same task.

**Results:**

German consonant cluster frequency had a significant facilitating influence on perception accuracy for both groups, which was even more pronounced for the L2 listeners. Conformity with the Sonority Sequencing Principle, on the other hand, had a significant inhibitory effect for both listener groups, contrary to expectations.

**Discussion:**

This suggests that it is experience with language-specific distributions that guides sublexical speech processing, also in an L2, while sonority sequencing does not play a facilitative role but rather seems to be correlated with a factor inhibiting successful recognition.

## 1 Introduction

Listeners employ several types of top-down information to make the speech perception process more efficient and error-resistant. Especially in noisy listening conditions, this can compensate for an insufficient bottom-up signal. On the sublexical level, top-down information includes the likelihood of different phoneme sequences. Whether this likelihood builds predominantly on language-specific distributions or is also informed to a considerable degree by universal phoneme sequencing regularities is not yet fully understood. Moreover, the relevance of these factors might vary between first- (L1) and second-language (L2) listening. This paper investigates how L1-German speakers’ perception of initial consonant clusters in noise is influenced, in addition to acoustic features, by the clusters’ frequency of use and by the degree to which they conform to sonority sequencing. In second-language perception, the general principles of L2 (i.e., target language) frequencies and L1 frequencies may differ. We further investigate their role in cluster perception. Given the critical role of word onsets for lexical access, this paper will focus on initial consonant clusters.

### 1.1 Language-specific phonotactics and usage-based theories of language

It is well-known that L1 phonotactics guides our perception (Finn and Kam, 2008; Weber and Cutler, 2006). Illegal consonant clusters, i.e., clusters disallowed by a language’s phonotactics, are prone to perceptual illusions: they are perceived as phonetically close legal clusters or an epenthetic vowel is inserted which is not there in the acoustic signal (Breen et al., 2012; Dupoux et al., 1999; see also Rossi et al., 2011, for evidence of neurological processing differences). Conceivably, this might also be true to a certain degree for low-frequency clusters: frequent consonant clusters should then be recognised more reliably than less frequent ones and the latter be mistaken for frequent ones more often, especially under uncertainty. This would then constitute an effect of gradient phonotactics, in contrast to effects of categorical phonotactics, namely legality.

Perceptual illusions of this type can be explained in terms of the mental representations of these clusters. Usage-based theories hold that our mental representations are shaped by language use. Representations of frequent structures are strengthened, and structures with stronger representations are in turn processed more easily and efficiently. For instance, frequent lexemes are recognised more reliably (Grosjean, 1980) and faster (Rubenstein et al., 1970) than infrequent ones. Low-frequency (LF) lexemes tend to be falsely recognised as high-frequency (HF) lexemes in situations of increased uncertainty (Felty et al., 2013; Grosjean, 1980). On the sublexical level, however, the available evidence is contradictory. In an identification experiment with synthetic liquid continua in consonant clusters of varying frequency, Pitt (1998) found no effects of cluster frequency beyond those of legality. He concludes that it is listeners’ knowledge of permissible phoneme sequences rather than frequency that is relevant for consonant cluster perception. Likewise, Cohen et al. (1967) found legality effects in both monolingual and bilingual populations, while a frequency-based account would leave many aspects of their data unexplained. In contrast, Hay et al. (2004) did find evidence of frequency effects on (heterosyllabic) clusters. They report that LF clusters were reanalysed more often than HF clusters and the direction of reanalysis was primarily from a less frequent to a more frequent cluster. They inferred that “[h]igh-frequency clusters attract responses, but only if they are acoustically similar to the speech signal” (Hay et al., 2004, p. 62). Lentz and Kager (2015) also found facilitation of frequent tautosyllabic (syllable-initial) consonant clusters in perception. These diverging results could be due to the types of consonant clusters tested or the task used, with the studies that used both legal and illegal clusters or identification tasks failing to find frequency effects.

In contrast to this conflicting evidence about the influence of consonant cluster frequency, phonotactic probability in general (i.e., calculated over the whole stimulus) has consistently been shown to have a facilitating effect on perception. Several studies found clear evidence that high phonotactic probability speeds up word recognition and makes it more accurate (e.g., Luce and Large, 2001; Vitevitch and Luce, 1998, 1999; Vitevitch et al., 1999). The role of consonant cluster frequencies in perception therefore deserves further investigation.

### 1.2 Universal preferences: sonority sequencing

Apart from frequency, an influence of universal structuring principles on speech perception has also been found, especially in the absence of distributional information in the language in question. Specifically, illegal phoneme sequences that violate the Sonority Sequencing Principle (SSP, Selkirk, 1981; Sievers, 1897) have been found to be more prone to misperceptions than illegal sequences that adhere to it (Berent et al., 2007; Moreton, 2002; Tamási and Berent, 2015). The SSP is based on the ordering of phoneme classes according to their inherent “loudness” (Ladefoged, 1975, p. 219) on a sonority scale or hierarchy. Each natural class of phonemes is assigned a sonority value which determines the class’s position on the scale. Various different scales (for an overview, see Parker, 2002) have been proposed, which differ in details, for example whether and how they subdivide obstruents and whether they subdivide vowels.^1^ A relatively fine-grained version of the sonority scale, which subdivides obstruents into stops and fricatives, is used by Berent et al. (2007), based on Goldsmith (1990):

(1) vowels > glides > liquids > nasals > fricatives > stops

          6               5             4              3                2               1

According to the SSP, the sonority values of segments in a syllable should increase from the onset to the nucleus and decrease thereafter. A more precise version is provided by the Sonority Dispersion Principle (SDP, Clements, 1990), which states that there should be a steep and even rise in sonority at the syllable onset and a gradual decline after the nucleus. These changes in sonority between adjacent phones can be expressed as sonority distances, which are calculated as pairwise differences between adjacent sonority values. For example, in the syllable /plaː/ the sonority distance between /p/ and /l/ is 3 (4–1, see scale in (1)) and the sonority distance between /l/ and /aː/ is 2 (6–4). The syllable is therefore well-formed in terms of the SDP, whereas /psaː/ with sonority distances of 1 and 4 would be less well-formed because there is only a slight sonority rise between the stop and the fricative. Sequences that deviate from the SSP and the SDP can cause perceptual illusions. For example, when English listeners are presented with the syllables /bzam/ and /bdam/, both of which are illegal in English, they are more likely to misperceive /bdam/, which constitutes a stronger violation of sonority sequencing. This means that language users’ preferences for syllables that are in line with the SSP and SDP, as shown in rating studies (e.g., Albright, 2007; Tamási and Berent, 2015), can also affect speech processing.

The effect does not seem to be purely auditory because onset clusters that violate sonority sequencing are also prone to misidentification in the visual domain (Tamási and Berent, 2015), suggesting that the preference of certain sequences over others and the resulting processing difference is found at a higher level. It seems to be restricted to unattested consonant sequences, however. Neurologically, there seems to be no difference in the processing of legal clusters that conform to the SSP and those that violate it (Deschamps et al., 2015). Some findings conflict with a sonority-based account. For example, Davidson and Shaw (2012) found significantly better recognition rates for fricative-initial clusters in syllable-initial position than for stop-initial clusters, while the opposite pattern was observed for final clusters by Bond (1971). From a sonority perspective, stop-initial clusters should be easier to perceive in syllable-initial position and fricative-initial clusters in syllable-final position. In addition, the participants in Davidson and Shaw (2012) often confused test clusters with clusters that were more marked, in other words, the perceptual repairs did not improve the markedness of the clusters but deteriorated it. The authors conclude that a sonority account does not hold when the differences in sonority between the consonants in a cluster are much smaller than in the cases tested by Berent and colleagues (see also Davidson, 2011). Instead, they attribute differences in perception accuracy to language-specific phonotactics. In L2 perception, however, universal sequencing principles such as the SSP might play a larger role as the target language is less entrenched and listeners are more likely to rely on other sources of top-down information rather than language-specific information.

### 1.3 Initial consonant clusters in German

German allows for a relatively large number of consonant clusters both in syllable-initial and in syllable-final position (see, e.g., Hanulíková and Dietrich, 2008, p. 123). Syllable-initially, 56 different consonant clusters consisting of up to three segments are attested (Orzechowska and Wiese, 2015). The largest group (n = 20) consists of an obstruent in the first position (C1) and a liquid in the second position (C2), e.g., /pl, tr, fl/. Other groups of initial clusters are obstruent–nasal (e.g., /kn, ʃn/; n = 10) and sibilant–obstruent (e.g., /ʃp, sf/; n = 9). Most of the clusters in the latter group are sibilant–stop clusters. Here, /ʃ/ is the more frequent sibilant that can be combined with any stop, nasal, or liquid except /k/^2^ and occurs in many words, among them some high-frequency lexemes, while /s/ can be combined with any stop, nasal, or liquid but occurs only in a limited number of words of foreign origin (e.g., slawisch /ˈslaːvɪʃ/, Spot /spɔt/). The composition of three-consonant clusters is limited to sibilant–stop–liquid, e.g., /ʃpl, ʃtr/.

In general, German onset clusters tend to conform to the SSP; almost 70% of them have a rising sonority profile (Orzechowska and Wiese, 2015). The largest group, namely the obstruent–liquid clusters, show a relatively steep rise in sonority from the stop with a value of 1 according to the scale in (1) above to the liquid with a value of 4. However, some of the most common German onset clusters are sibilant–stop clusters (e.g., the single most frequent cluster /ʃt/), which present a violation of the SSP: here, sonority decreases from the sibilant (i.e., fricative; sonority value = 2) in C1 position to stop in C2 position (sonority value = 1).^3^ These clusters are prominent SSP violations in many languages. For a full discussion of several proposals of how to treat them in phonological theory, see Goad (2011).

It is worth noting that there is some dialectal variation regarding consonant clusters due to different phonological processes being operative in the different dialects. This concerns both the maximal syllable structure, the frequency of occurrence of individual clusters, and the distribution in terms of sonority dispersion (see Lameli, 2022, for an exhaustive discussion). For example, the perfect participle prefix *ge*- is typically shortened to /g/ or /k/ by schwa apocopation in Southern German dialects and regional varieties, thus creating a consonant cluster with the initial consonant of the following verb root. (e.g., *gesagt* /gə’zakt/ > /ksakt/ ‘said’). This greatly increases the frequency of clusters like /ks/ or /gm/ in these dialects, which have a very low type frequency in Standard German. Northern German dialects, on the other hand, feature lenition of /g/ to /j/ (Lameli, 2022), effectively turning stop–liquid clusters into glide–liquid clusters.

### 1.4 L2 perception

Perception of an L2 differs in several ways from perception of the L1. On the one hand, this difference relates to phonetic factors and on the other hand to higher-level factors, such as lexical and phonological knowledge. On the phonetic level, L1 listeners use multiple, redundant acoustic cues and cue weighting to ensure successful phoneme identification also under adverse listening conditions. In contrast, L2 listeners might use different cues and weighting strategies, which are often influenced by their L1 (Davidson and Shaw, 2012; Lecumberri and Cooke, 2006). In noise, the richness or paucity of the cues used can be decisive. The cues that L2 listeners usually attend to may be masked, while cues additionally employed by L1 listeners may withstand masking and still be available. Therefore, noise-masking affects L2 listeners more than L1 listeners (Lecumberri and Cooke, 2006). On the other hand, an electrophysiological study found that L2 listeners attend more to acoustic detail than L1 listeners (Song et al., 2019). This strategy might compensate for some of their difficulties, albeit only for phonemes with clearly perceptible cues.

On a higher level, L2 listening is distinct from L1 listening because the top-down knowledge differs between L1 and L2 listeners. Most importantly for sublexical processing, the phonotactic knowledge differs. Firstly, L2 listeners do not have as much experience with the phonotactic distributions of the target language as L1 speakers. The question is therefore whether they can use their knowledge of the target language in the same way as L1 listeners can. Secondly, their L1 phonotactics might interfere with L2 processing. Hence, it is not clear how L1 and L2 phonotactics interact during L2 processing. Previous studies on L2 processing have mostly analysed phonotactics in a categorical sense, distinguishing between legal and illegal sound sequences. They have shown that listeners apply both L1 and L2 phonotactic knowledge during L2 speech segmentation (Hanulíková et al., 2011; Weber and Cutler, 2006) and L2 speech perception (Carlson, 2018; Carlson et al., 2016; Ulbrich and Wiese, 2018). The fact that knowledge of two phonotactic systems can be integrated and jointly influence speech perception also shows in perceptual repairs. Here, the type of perceptual illusions described in Section 1.2 diminish with the acquisition of another, less restrictive, phonotactic system (Carlson, 2018). Only a small number of studies fail to find effects of at least one type of phonotactics: either L1 or L2. Trapman and Kager (2009) found that L2, but not L1, phonotactic knowledge affected learner performance in a lexical decision task involving onset clusters, and in the case of a more restrictive L1, it only affected advanced learners. In contrast, misperceptions of L2 ambisyllabic consonant clusters were not influenced by the legality of the cluster in the L1 in a study by Kabak and Idsardi (2007). In addition to categorical phonotactics, which refers to the (il-)legality of sequences of segments, phonotactics can also be viewed in a gradient sense, namely as the relative frequencies of different clusters. A few studies suggest that gradient phonotactics plays a role during different speech processing tasks. For example, Slovak learners of German were to a larger degree influenced by their L1 frequencies of initial consonant clusters when segmenting German speech than by the L2 frequencies (Hanulíková et al., 2011). At the same time, they were able to apply rule-based segmentation strategies that are specific to the L2 and do not hold for Slovak speech segmentation, which shows that they are able to make use of properties of the L2 phonological system. Lentz (2011) also found evidence that L1-Slavic learners of Dutch use the gradient phonotactic knowledge of their L1 for L2 segmentation. However, it was not possible to completely disentangle the influence of gradient L1 phonotactics and (partly acquired) categorical L2 phonotactics. Finally, Lentz and Kager (2015) tested for both gradient effects of L2 phonotactics and categorical effects of L1 phonotactics and found both to be present simultaneously: L2 gradient phonotactics is acquired but via an L1 phonotactic filter, which causes perceptual vowel epenthesis in L1-illegal consonant clusters. Their participants learned which consonant clusters are frequent and which ones are infrequent in the L2 and were able to use this knowledge during speech processing. However, their representations of L2-frequent and less frequent consonant clusters were not faithful but were corrected according to the phonotactic rules of their respective L1s. This means that L2 learners filter sound sequences through the eye of the L1, much like individual sound categories are filtered through it (Archibald, 1998; Best and Tyler, 2007; Trubetzkoy, 1939). These studies indicate that while L2 listeners can use L2 statistical knowledge for speech processing, they are also influenced by the structural properties of their L1.

Universal principles, such as sonority sequencing, may be more relevant to L2 perception than L1 perception. Indeed, it has been shown that the SSP-conformity of consonant clusters has an effect on nonce word recall (both for learners with a phonotactically less restrictive and a phonotactically more restrictive L1) which was stronger than that of L2 legality (Ulbrich and Wiese, 2018). In contrast, L1-Russian learners of Dutch were not influenced by sonority-based well-formedness in a lexical decision task, although they showed sensitivity to sonority sequencing in wordlikeness ratings (Trapman and Kager, 2009). The latter speaks against a greater role of sonority in L2 processing than in L1 processing.

In summary, there seems to be some sensitivity to sonority in the processing of L2 sequences, but it does not surface during all speech processing tasks and in all measured variables. Generally, sonority sequencing seems to be more influential in meta-linguistic and recall tasks than in online processing.

### 1.5 Present study

In the study reported here, the influences of both higher-level factors (language-specific distributions of initial consonant clusters and sonority sequencing) on sublexical speech processing were compared, while acoustic factors were also taken into account. The aim was to explore the relative contributions of language-specific and universal factors in L1 and L2 perception. We used 16 legal German initial consonant clusters of varying frequency, four of which violate the SSP, in an identification-in-noise task with German listeners and Australian learners of German. The noise, specifically multi-talker babble as a very naturalistic type of noise, was added to avoid ceiling effects.

We predicted that due to listeners’ experience with language-specific distributions, HF consonant clusters are processed more accurately than LF clusters and at the same time serve as defaults for perception in cases of uncertainty. The following hypotheses are derived from these assumptions:

Hypothesis 1: The error rate decreases with higher frequency of a consonant cluster.

Hypothesis 2: Perceptual repairs will mainly result in HF consonant clusters.

We also predicted that consonant clusters conforming to the universal principles of sonority sequencing should be better processed than those which do not.

The following hypotheses are derived from this assumption:

Hypothesis 3: Consonant clusters that violate the SSP will have higher error rates than those that conform to it.

Hypothesis 4: Misperceptions should improve the sonority profile of the cluster and will therefore tend to result in the illusionary perception of a cluster of a greater sonority distance.

In line with usage-based theories, which stress the role of experience and frequencies of use in shaping mental representations and guiding speech processing (cf. Bybee and McClelland, 2005; Ellis, 2002), we predicted that cluster frequency plays a greater role than the universal SSP.

## 2 Experiment 1: L1 perception

### 2.1 Materials and methods

#### 2.1.1 Participants

Thirty-five L1-German speakers (22 female, mean age: 24.06, SD = 4.10), mostly students at the University of Freiburg, were tested and paid for their participation. None of them reported any hearing impairment and none of them had grown up in the south of Germany. The call for participation explicitly ruled out dialect speakers and speakers from the south of Germany to avoid systematic frequency discrepancies for the cluster /ks/ due to dialectal schwa apocopation (see Section 1.3 above). All participants gave written informed consent and were free to terminate their participation at any time.

#### 2.1.2 Materials

##### 2.1.2.1 Stimuli

Out of the 56 legal German onset clusters mentioned in Section 1.3, 16 were chosen to be used in pseudowords so that they vary in frequency of use, cover different classes of clusters (e.g., stop–liquid, fricative–nasal, sibilant–stop), and can be paired up either as a minimal pair differing in one phonological feature (e.g., /ʃp/ and /sp/) or with a cluster consisting of the same consonants in reversed order (e.g., /sk/ and /ks/). The latter criterion was only relevant for a speech production experiment (Wulfert et al., 2022), for which we selected the same 16 clusters to achieve the best comparability across modalities and tasks Only attested clusters were used for a valid comparison of the influence of language-specific statistics and sonority sequencing. Table 1 lists all clusters selected along with their frequencies of use and SSP status as well as mean intensity and duration of the stimulus tokens used in the experiment. All frequency information is based on log-transformed position-specific type frequencies, i.e., the number of unique lemmas in the German lexicon that contain the consonant cluster in syllable-initial position, taken from the WebCELEX database (Max Planck Institute for Psycholinguistics, 2001). Since the cluster pairings are not relevant to the present study, we list the clusters individually in Table 1 for better readability. For each test cluster, 10 monosyllabic pseudowords were created, resulting in 160 stimuli with initial consonant clusters. Pseudowords rather than real words were used in order to avoid lexical effects; for the same reason, they were lexically opaque (cf. Raettig and Kotz, 2008). All Standard German vowels, including diphthongs, (/a, aː, eː, ɛ, iː, ɪ, oː, ɔ, uː, ʊ, øː, œ, yː, ʏ, aɪ, aʊ, ɔʏ/) were used as nucleus vowels and all possible singleton coda consonants (/p, t, k, f, s, ʃ, ç, x, m, n, ŋ, l, r^4^/) were used as coda consonants. They were distributed as evenly as possible across the stimuli, over onset clusters, and over experimental blocks. All test stimuli had the form CCVC (with long or short vowel or diphthong, e.g., /ʃteːm/, /flœp/, /ksaɪn/). To ensure that none of the pseudowords is an actual German word, phonemic transcriptions were compared against those in the German CELEX database.^5^ In addition to the 160 test stimuli, 100 filler stimuli (also monosyllabic pseudowords) were created, 73 of which had simple onsets. The remaining 27 filler items started with consonant clusters that were not part of the test set (e.g., /kluːf/, /ʃvøːt/) and were included in order to reduce cues to the identity of the test clusters and hence avoid biases towards reporting them. We did not treat them as target clusters because the number of items per cluster was too low and their distribution too uneven to provide systematic additional information for the analyses.

**Table 1:** haracteristicsof the 16 test s, liste in descending order of frequency.

Cluster	Frequency	Natural class	SSP status	∅ duration	∅ intensity
/ts/^a^	3.25	stop–fricative	no violation	137 ms	62.31 dB
/ʃt/	3.16	fricative–stop	violation	277 ms	58.86 dB
/ʃp/	2.91	fricative–stop	violation	274 ms	55.37 dB
/tr/	2.88	stop–liquid	no violation	112 ms	57.28 dB
/kr/	2.61	stop–liquid	no violation	106 ms	57.31 dB
/ʃl/	2.54	fricative–liquid	no violation	244 ms	64.13 dB
/fl/	2.40	fricative–liquid	no violation	224 ms	64.92 dB
/ʃm/	2.25	fricative–nasal	no violation	266 ms	65.69 dB
/pl/	2.23	stop–liquid	no violation	123 ms	64.12 dB
/ʃn/	2.18	fricative–nasal	no violation	266 ms	66.31 dB
/sk/	1.94	fricative–stop	violation	284 ms	58.68 dB
/ps/	1.54	stop–fricative	no violation	160 ms	60.08 dB
/sl/	1.36	fricative–liquid	no violation	245 ms	64.81 dB
/tʃ/^b^	1.11	stop–fricative	no violation	122 ms	61.24 dB
/ks/	0.95	stop–fricative	no violation	137 ms	60.71 dB
/sp/	0.85	fricative–stop	violation	276 ms	57.70 dB

Frequencies are log-transformed CELEX type frequencies. Onset durations and onset intensities are averaged over all ten stimuli for a given cluster.

a The complex onset $/\overset{⌢}{\mathrm{ts}}/$ is considered an affricate in German phonology. Nonetheless, it has been included in the test set because of its strong structural similarity to the true clusters /ps/ and /ks/, from which it differs greatly in terms of frequency. This biphonemic analysis is in line with Heike (1972), for example.

b /tʃ/, which occurs in words of foreign origin like ‘Cello’ (‘cello’), ‘tschechich’ (‘Czech’), ‘checken’ (‘to check, to understand’) as well as the onomatopoetic ‘tschilpen’ (‘to chirp’), is not considered an affricate in Standard analyses of the German phonological system (e.g., Trubetzkoy, 1939; but see Hall, 1992, for a different view).

The stimuli were spoken by a trained female L1 speaker of German in standard pronunciation, which entailed a fricative-like articulation of /r/ in the clusters /tr/ and /kr/. In line with standard analyses of the German phoneme inventory and phonotactics (e.g., Hall, 1992; Wiese, 2003), we decided not to treat /r/ as a fricative sonority-wise in spite of its phonetic realisation as the voiced uvular fricative [ʁ]. However, since /tr/ and /kr/ conform to the sonority hierarchy both when regarding /r/ as a fricative and when regarding it as a liquid/an approximant, this does not change anything about our classification of clusters in terms of SSP conformity and hence nothing about our SSP analyses, either. The only change would be in the sonority distance value of /tr/ and /kr/ (from 3 with /r/ as a liquid to 1 as a fricative). We will return to this point in the Discussion. The stimuli were recorded in a sound-attenuated booth with an AKG C2000B microphone in Adobe Audition. They were recorded in stereo channel with a sampling rate of 44,100 Hz. Each stimulus item was spoken at least three times and the best token of each item was selected.^6^ Criteria for token selection were a clear articulation of all phones and a similar prosody and speaking pace across all stimuli. All stimuli were RMS-normalised to 65 dB Sound Pressure Level (SPL) in Praat (Boersma and Weenink, 2018). As this normalisation relies to a large part on the vowel of the stimulus, the onsets were only approximately equal in intensity. Both intensity and duration were significantly different for some of the clusters (see Table 1), as is to be expected in light of the natural variation between consonant classes.

##### 2.1.2.2 Multi-talker babble

Twenty recordings of German audio books (10 male and 10 female readers) from a public domain^7^ served as the source for the multi-talker babble. The optimal number of babble talkers was determined by auditory checking of different variants until the babble sounded uniform and almost no individual words could be recognised. The 20 recordings were chosen based on audio quality, clarity of pronunciations and steady intonation, and were processed as follows: (1) removal of silences above 0.15 s in Praat, (2) RMS normalisation to 65 dB SPL, (3) mixing of individual files in Audacity software (Version 2.1.1^8^), (4) trimming to length of the shortest source file (to ensure that all talkers were present at any given time) and exclusion of the initial 15 s, which contained general announcements and the title, (5) cutting into pieces of 2 s, (6) final normalisation of babble pieces to 64 dB SPL, and (7) auditory checking of all babble pieces and elimination of pieces with noticeable intensity peaks. This process resulted in 419 different pieces of babble, each 2 s long and containing speech from all 20 speakers. Normalising the stimuli to 65 dB SPL and the multi-talker babble to 64 dB SPL yielded a signal-to-noise ratio (SNR) of +1 dB SPL averaged over the whole stimulus. The actual difference in intensity between signal and noise at stimulus onset (which is the sole determiner of accuracy) varied. Mean intensity of the stimulus onsets was 61.22 dB SPL (leading to a mean SNR of −2.78 dB SPL at syllable onsets), with a standard deviation of 3.48. The SNR is based on observations from a pre-test with four participants, none of whom participated in the final experiment.

#### 2.1.3 Procedure

Before the experiment, all participants completed a language background questionnaire, which is provided in Supplementary materials. They were seated one at a time in a sound-attenuated booth and equipped with headphones. The task was an open-set recognition, i.e., a free transcription of the stimuli. The 100 filler items were used to ensure that participants would not recognise the task as closed-set recognition and only used answers from the set of test clusters. The experiment was run in OpenSesame 3.1.9 (Mathôt et al., 2012) on a MacBook Pro. It was divided into five blocks of 52 stimuli each. After each block, a screen informed participants that they had completed the nth block, and instructed them to take a short break and then press the enter key when they were ready for the next block. The order of the blocks was counterbalanced across participants using a Latin Square design, and the order of the stimuli within each block was pseudo-randomised by the experiment software. Pseudo-randomisation included the following constraints: (1) No test cluster can appear in two consecutive trials. (2) Not more than three test items or three filler items can appear in succession.

Participants were instructed orally and in written form to listen carefully to the nonsense syllables in babble and type exactly what they heard when they saw the prompt on the screen. Participants could correct their responses before pressing the enter key and initiating the next trial. The experiment was thus fully self-paced. In addition, they obtained and kept a sheet showing the desired spelling system by means of examples (e.g., <s-p> to denote /sp/ because <sp> is conventionally used to denote /ʃp/ in German orthography). Audio examples of possible stimuli (without noise) were given along with specification of the desired spelling.

Before the syllable transcription task started, a simple hearing screening of 15 pure tones at five different frequencies (500, 1,000, 2000, 4,000, and 8,000 Hz) at the lowest amplitude possible (approximately 25 dB SPL) was administered to ensure that none of the participants had an acute or permanent hearing impairment.

Ten practice trials were used to familiarise participants with the task and the desired spelling of the nonce syllables. Participants received feedback on the computer screen, with the correct answer spelled out. Participants were encouraged to set the volume at a comfortable listening level during the practice trials and leave it constant throughout the experiment. During each trial, the stimulus was played only once and could not be repeated. Multi-talker babble started 415 ms before the onset of the stimulus and continued after the offset of the stimulus for a total of 2 s. Each stimulus was randomly assigned to one of the babble segments for every participant anew to minimise the effect a given babble segment may have on a stimulus. The whole session lasted about 45 min.

#### 2.1.4 Analysis

Errors in the onset consonant clusters were coded in a binary fashion: Onsets were transliterated into phonetic writing (specifically, SAMPA for machine readability to make automatic comparison of target and typed response possible) and compared to target onsets. It turned out that /sp/ was spelled <sp> (which was meant to denote /ʃp/, see above) instead of <s-p> more often than hearing errors could plausibly account for. This appears to be attributable to difficulties in adhering to the spelling instructions set up for the experiment. The number of errors for /sp/ was therefore disproportionately high. Hence, all cases in which the stimulus cluster /sp/ was transcribed as <sp> had to be excluded from the analysis (thereby probably also eliminating some cases of true hearing errors). Moreover, two cases had to be excluded from the analysis because they contained empty responses. After this procedure, 5,452 out of the original 5,600 observations were left in the data set (exclusion rate: 2.64%).

To test hypotheses 1 and 3, the data were analysed in a logistic mixed-effects regression model using the *glmer* function of the *lme4* package (Bates et al., 2015) in R (R Core Team, 2016). Perception error in the onset served as the dependent variable. Log cluster frequency (based on CELEX type frequencies), and SSP violation (categorical variable with the levels *no violation* and *violation*) were entered as fixed effects. We used the sonority scale in (1) on p. 3 to determine violations of the SSP because, in contrast to the sonority scale proposed specifically for German by Hall (1992), it distinguishes between stops and fricatives, which is essential for a comparison of stop–sibilant vs. sibilant–stop clusters. We further added consonant cluster intensity and duration as control variables to account for the inherent intensity and duration differences between the different types of cluster used (cf. Table 1).

Since consonants with different manners of articulation vary inherently in intensity and duration, we first tested possible correlations between Sonority Distance (which ranks different cluster types on an ordered scale ranging from −1 for fricative–stop clusters and 3 for stop–liquid clusters) and intensity as well as Sonority Distance and duration. The correlation was weak for intensity (*r*_s_ = 0.28, *p* < 0.05) but strong for duration (*r*_s_ = −0.65, *p* < 0.001). These correlations are still low enough—also for duration—for these terms to be informative when included in the model, even though there is inherent variability between cluster classes. For illustrative purposes, we plotted intensity and duration of the different clusters, grouped by Sonority Distance (see Supplementary Figures 1, 2).

Based on the fact that sonority effects are usually observed for unattested clusters, it seemed plausible that frequency and SSP violation could interact, which might result, for example, in stronger sonority effects for LF than for HF clusters. Therefore, an interaction term between the two predictors was also included. The random structure included random intercepts by subject and by item (the latter nested under onset cluster) as well as random slopes for frequency, SSP violations, and their interaction. Predictors that did not show a significant effect were then removed in a stepwise fashion and models compared using the *anova* function in R until the model that best explained the data was found. The numerical variables were centred before being entered into the model. For the categorical variable SSP violation, sum coding was used.

To test Hypothesis 2, frequencies of the target clusters were compared to those of the clusters reported by the listeners. The data was subsetted to include only error trials in which the percept constituted a legal German CC or CCC onset cluster, which also includes clusters not in the set of target clusters. To test Hypothesis 4, sonority distances were compared between target and reported clusters in parallel fashion. Here, the subset of percepts was limited to legal CC clusters so that there was only one consonant–consonant transition.

### 2.2 Results

#### 2.2.1 Accuracy

**Figure 1:**
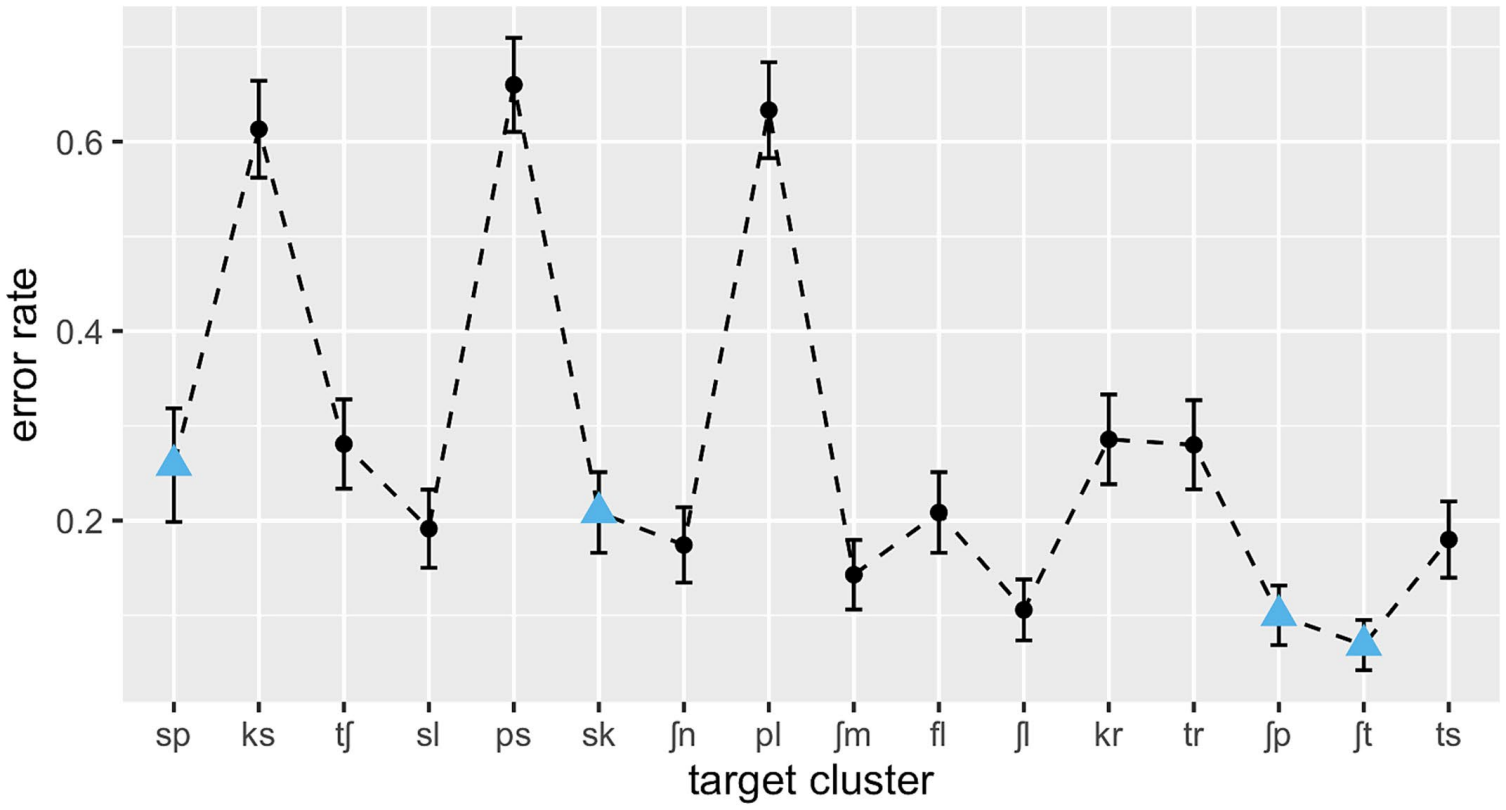
L1 error rates over consonant clusters in ascending order of type frequency; SSP-violating clusters are marked as blue triangles.

The overall recognition of onset clusters was above chance level, with an overall error rate of 27.5% (SD = 0.32). Performance varied widely, however, both between subjects (error rates ranging from 17.9 to 47.5%) and between consonant clusters, with an error rate of 6.8% for the most perceptible cluster (/ʃt/) and one of 66.0% for the hardest cluster to recognise (/ps/). Figure 1 shows the mean error rates for the individual clusters across subjects.

##### 2.2.1.1 Logistic regression

**Figure 2:**
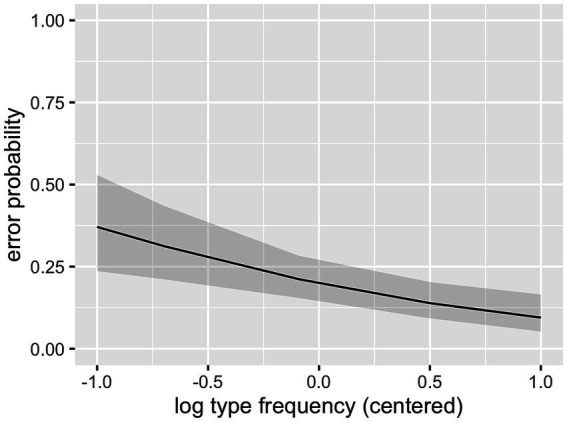
Effect of cluster frequency (L1 data).

**Figure 3:**
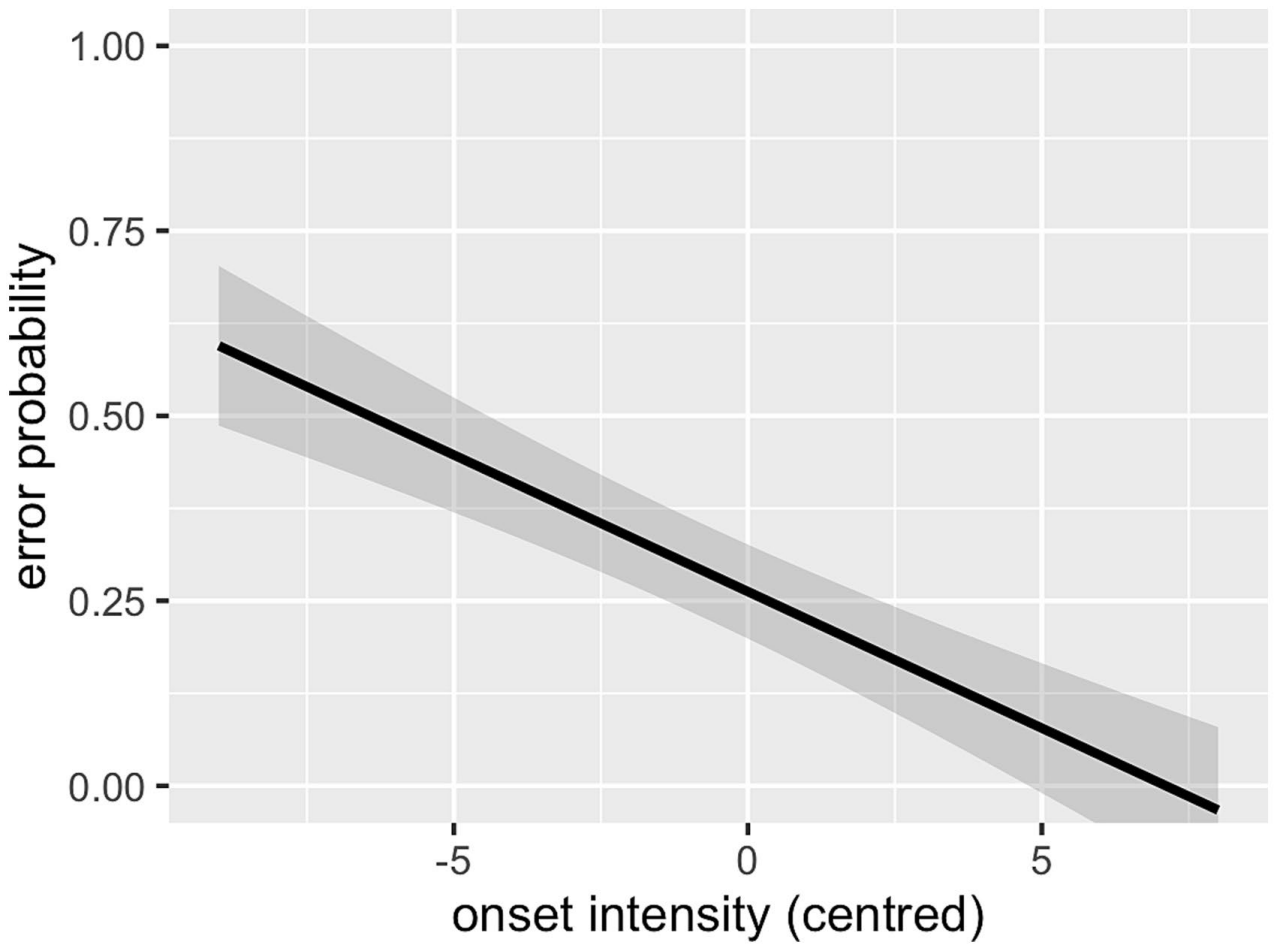
Effect of onset intensity (L1 data).

**Figure 4:**
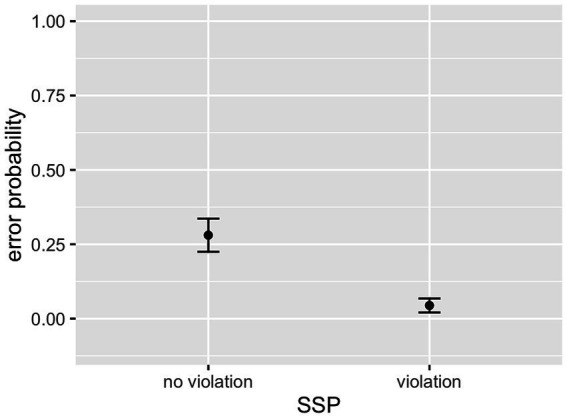
Effect of SSP violation (L1 data).

As can be seen in Table 2, the interaction between cluster frequency and SSP violation did not reach significance. In line with our prediction, there was an effect of log cluster frequency, with higher frequency resulting in lower error rates (Figure 2), and an effect of onset intensity, with clusters of higher intensity causing lower error rates (Figure 3). The effect of SSP violation is not in line with our hypothesis, because consonant clusters that violate the SSP were perceived better than those that do not (Figure 4).

**Table 2:** Summary of L1 perception logistic regression model.

Fixed effects				
Effect	β	*SE*	*z*	*p*
(Intercept)	−2.012	0.265	−7.587	<0.001
Onset intensity	−0.236	0.041	−5.821	<0.001
Cluster frequency	−0.796	0.303	−2.629	0.009
SSP violation	1.128	0.270	4.182	<0.001
Cluster freq × SSP violation	0.004	0.300	0.012	0.991
Random effects				

Effect	Variance	SD
Item:target cluster (Intercept)	0.899	0.948
Subject (Intercept)	0.188	0.433
Cluster freq (log)	0.165	0.406
SSP violation	0.049	0.222
Cluster freq × SSP violation	0.115	0.338
Target cluster (Intercept)	0.599	0.774
Marginal *R*^2^ = 0.186; Conditional *R*^2^ = 0.486		

Formula: error ~ons.intensity + logFreq * SSP.vio + (logFreq * SSP.vio|subjID) + (1|onset.targ/stimulus).

#### 2.2.2 Analysis of perceptual repairs

The majority of misperceptions constituted legal German onsets; only 5.9% violated German phonotactics (e.g., /tsl, spf/). The most common type of perceptual error varied between target clusters. While for some stop-initial clusters the most common confusion was between places of articulation of the stop (e.g., /ps/ and /ks/ reported as /ts/, /pl/ reported as /kl/), /kr/ and /tr/ showed mostly voicing errors on the stop, and for /ts/ and /tʃ/ deletion of the stop was most common. A complete confusion matrix can be found in Supplementary Table 1.

##### 2.2.2.1 Frequency comparison

According to Hypothesis 2, perceptual repairs should mainly result in HF consonant clusters. The frequency distribution of the reported clusters supports this hypothesis. By far the most common outcome of a misperception was /ts/ (resulting mainly from misperceptions of /ps/ and /ks/), the onset with the highest frequency. In general, a trend can be identified for the number of false positives to increase with the frequency of a cluster. Moreover, the number of misperceptions directed towards higher frequency was almost twice as high as that directed at lower frequency (see Table 3). The direction of misperceptions thus constituted a strong trend but not an absolute rule.

**Table 3:** Frequency comparison of clusters (target vs. cluster reported by listener) in misperceptions.

Frequency comparison	Number of observations
frequency of cluster reported by listener > frequency of target cluster	559 (37.32%)
frequency of cluster reported by listener < frequency of target cluster	334 (22.30%)
other (listener reported perceiving illegal CC or CCC)	605 (40.39%)

##### 2.2.2.2 Comparison of sonority distances

In contrast to what was expected, most misperceptions preserved the sonority distance between the two consonants in a cluster (see Table 4). Only a small minority improved the sonority profile of the onset, while more than twice as many deteriorated it. Hence there is no trend in misperceptions to increase the sonority distance in a cluster. For many misperceptions the sonority distance of the reported onset was not determinable, for example because it does not have the format CC.

**Table 4:** Comparison of sonority distances in target clusters vs. clusters reported by listener.

Comparison of sonority distances	Number of observations
sonority distance_reported cluster_ > sonority distance_target_	110 (7.34%)
sonority distance_reported cluster_ < sonority distance_target_	252 (16.82%)
sonority distance_reported cluster_ = sonority distance_target_	580 (38.72%)
sonority distance_reported cluster_ not determinable	556 (37.12%)

Unsurprisingly, the acoustic make-up of a cluster played a significant role for its identification. Onset intensity had a significant facilitating effect on identification accuracy. Another acoustically conditioned pattern in the data is that stop-initial, and especially stop–sibilant, clusters were misperceived far more often than other classes (see below for discussion).

### 2.3 Discussion

#### 2.3.1 Cluster frequency

As predicted, high frequency of a consonant cluster facilitated its recognition in noise. The frequency effect becomes especially evident when clusters of similar composition have divergent recognition rates (as is the case for /ʃp/ vs. /sp/) or when the same clusters show divergent recognition rates depending on the language background of the listener. In a comparable^9^ perception study with Italian and Dutch listeners (Baroni, 2014), /ʃt/ and /ʃp/, which are both phonotactically illegal in Italian and Dutch, showed relatively high error rates (56 and 38%, respectively). Conversely, /sp/ and /sk/, which are legal in Italian and Dutch, had very low error rates (1.6 and 4.7%, respectively). While the phonetic characteristics of the clusters are the same in both experiments, their phonotactic status differs in the languages tested, and this difference is mirrored in the error rates. This means that under the adverse listening conditions, listeners are biased by their structural knowledge to perceive the consonant clusters that are most expectable in the respective language.

Baroni (2014) interprets the results of his study in terms of absolute phonotactics, differentiating merely between legal and illegal clusters. However, the error rates of the present experiment show the gradience of the effect: the HF clusters /ʃt/ and /ʃp/ have the lowest error rates among the sibilant–stop clusters, LF /sp/ has the highest error rate, and /sk/ ranks in between the two, just as its intermediate frequency rank would lead one to expect. Statistically, the gradience is underpinned by the significant effect of the numeric predictor frequency. The German preference for /ʃ/−initial clusters is also reflected in the low error rate of /ʃl/ as compared to /sl/. Again, that corresponds to the lexical distribution of these clusters. In Dutch or English listeners, the opposite pattern would be expected. Similarly, the strong divergence in stop–sibilant error rates (61 and 66% for /ks/ and /ps/, respectively, and 18% for /ts/) corresponds remarkably well to their frequency difference.

It could be argued that the extraordinarily good perceptibility of $/\overset{⌢}{\mathrm{ts}}/$ can be ascribed to its phonological status as an affricate, which distinguishes it from /ks/ and /ps/, rather than its high frequency of use as a German onset. Two reasons speak against this interpretation. First, looking at the error rates of filler items in the experiment starting with the affricate $/\overset{⌢}{\mathrm{pf}}/$, whose CELEX type frequency is about a tenth that of $/\overset{⌢}{\mathrm{ts}}/$, indicates that affricate status does not by itself guarantee accurate perception: the two filler stimuli beginning with $/\overset{⌢}{\mathrm{pf}}/$ (summing up to 70 observations across participants) showed an error probability of 80%. Second, the listeners’ frequent perceptual repairs of /ks/ and /ps/ to /ts/ (see next paragraph) testify to the fact that they perceived them as structurally equivalent. Although $/\overset{⌢}{\mathrm{ts}}/$ (as well as $/\overset{⌢}{\mathrm{pf}}/$) differs in its phonological status from /ks/ and /ps/, their phonetic status is the same. Interestingly, the same pattern was reported in a study on the L1 acquisition of initial /s/−stop and stop−/s/ sequences in Greek: there was no difference in accuracy between $/\overset{⌢}{\mathrm{ts}}/$, which is considered an affricate in Greek as well, and the clusters /ks/ and /ps/, while all of them had lower accuracy rates than /s/−stop clusters (Syrika et al., 2011). We therefore argue that although $/\overset{⌢}{\mathrm{ts}}/$ phonologically is usually considered an affricate in German, this status depends on the theoretical framework (linear vs. non-linear) and does not seem to impact behavioural results. It is therefore legitimate to group it with the true clusters /ks/ and /ts/.

Turning to the outcomes of misperceptions, it is obvious that cluster frequency played a role here, too. Generally speaking, the higher the frequency of a cluster, the more often it was reported as perceived instead of the target cluster. This is particularly obvious in the case of /ts/ (the onset with the highest type frequency in the test set), which attracted a high number of responses from the LF target clusters /ps/ and /ks/. Target /ts/, on the other hand, is hardly ever perceptually repaired to /ps/ or /ks/, so the confusion is asymmetric. It is also worth noting that HF /kr/ was not perceptually repaired to higher-frequency /tr/ very often. Hence, in phonologically similar pairs of consonant clusters, the direction of confusion is clearly biased towards the HF cluster, and this bias is strongest when there is a large frequency difference between the clusters. This even leads to perceptual asymmetries between two phonemes being reversed when the frequency relations of the clusters that they appear in are reversed. For example, the perceptual illusion /s/ > /ʃ/ is very common in sibilant-initial clusters (hereafter referred to as sC clusters), while the opposite is not true. For /ts/−/tʃ/, on the other hand, the confusion goes in the opposite direction (/ʃ/ > /s/), again turning a LF cluster into a HF one. Asymmetries in perceptual confusions have been observed before and have previously been attributed to acoustic–phonetic factors, such as the energy profile of the consonants involved (Chang et al., 2001; Moreno-Torres et al., 2017) and the phonetic context (Woods et al., 2010), to phonological factors, such as phonological underspecification of phonemes (Lahiri and Reetz, 2002, 2010), and to higher-level factors such as phoneme and lexical frequency as well as phonological neighbourhoods (Benkí, 2002, 2003; Moreno-Torres et al., 2017).

The phonotactic effect in the present data is not limited to illegal onset structures. Participants experienced perceptual illusions—not in the form of epenthetic vowels, as often induced by studies on illegal clusters (e.g., Berent et al., 2007; Carlson, 2018; Lentz and Kager, 2015), but in the form of cluster confusions and reductions—for low-frequency clusters. It can therefore be concluded that phonotactic knowledge is gradient, or at least that a gradient form of phonotactic knowledge exists (possibly alongside a categorical one).

There are also studies, however, whose results diverge from the present findings. For example, in a gating experiment with all legal Dutch biphones, Warner et al. (2005, p. 70) found only a weak influence of phoneme frequency on listeners’ responses and no significant influence of transitional probabilities between the two phonemes and conclude that “listeners can do quite well […] at recognizing individual sounds, from bottom-up information alone. Listeners certainly do not have to rely on higher-level information such as overall frequency or transitional probabilities in order to decide what sounds they are hearing.” In a similar vein, Pitt (1998) found no effect of consonant cluster frequency beyond that of legality in perception experiments with synthetic consonant continua. While it is certainly true that listeners can recognize phoneme sequences from the bottom-up signal alone in quiet listening conditions (as was the case in the gating experiment), the present results show that they can also make use of their statistical knowledge of the language and are very likely to draw on this resource when the bottom-up signal is less reliable. This is in line with the conclusion by McQueen and Pitt (1996) that probability effects in auditory perception occur mainly when the information in the audio signal is reduced.

#### 2.3.2 Sonority sequencing

In contrast to our prediction, sonority sequencing did not have a facilitating effect on processing. On the contrary, clusters violating the SSP had a significantly lower error probability than the ones conforming to it. Moreover, most misperceptions did not improve the sonority profile of a cluster. This is unexpected in light of phonological accounts featuring sonority as a linguistic principle relevant to both language structure and change and psycholinguistic processes such as language acquisition (e.g., Gómez et al., 2014; Yavaş, 2003). It is also in conflict with the empirical evidence on the role of sonority sequencing in speech processing reviewed in Section 1.2. Hence, it must be assumed that there is no true inhibitory effect of SSP conformity in the present data but that it is an artefact of SSP conformity/violation being confounded with another variable—or a combination of variables—which cause(s) the effect. For example, SSP violation is positively correlated with onset duration (*r_pb_* = 0.60, *p* < 0.001), which failed to show an effect in the model. However, it is clear that there was no *facilitative* influence of SSP conformity. These results deviate from those by Tamási and Berent (2015), who found a facilitative effect of sonority distance on perception, and a consonant cluster rating study by Albright (2007). The most obvious difference between the studies is that we used only attested consonant clusters (in some cases marginally attested but nonetheless legal), i.e., clusters occurring in German lexemes. In contrast, Tamási and Berent (2015) used solely unattested clusters and Albright (2007) attempted to develop a model that accounts for well-formedness judgements of both attested and unattested consonant clusters. An intuitive explanation for the diverging results is therefore that prior biases can be overridden by statistical learning and are thus only visible where it does not apply. Note that there was no significant interaction between cluster frequency and SSP violation, thus providing no indication that listeners resorted to sonority to guide the listening process in the cases of uncommon clusters. Interestingly, Albright (2007, p. 4) only used stop-initial clusters and thus did not run into conflicts with sC clusters. In contrast, in an fMRI study involving auditory and visual presentation of pseudowords that did include sC clusters, Deschamps et al. (2015) did not find an effect of sonority in the auditory modality and conclude that it is absent in auditory processing (p. 82). Hence, the presence or absence of a sonority effect seems to depend on the legality status of the consonant clusters and potentially also on the inclusion of sibilant–stop clusters in the test set. In the present experiment, the inclusion of both sibilant–stop and stop–sibilant clusters most likely caused the apparent anti-sonority effect, in line with Davidson and Shaw (2012) and Bond (1971).

In light of the unexpected null results in the present study it might also be asked whether the binary sonority measure distinguishing only between SSP-adhering and SSP-violating clusters is too coarse to show fine-grained, meaningful sonority effects. To test for this possibility, exploratory logistic regression models were set up *post hoc*, which were identical to the model reported above except for the fact that the binary predictor SSP violation was replaced by sonority distance, a factor with several levels (in the first post-hoc model: −1 for SSP-violating clusters and 1–3 for SSP-conforming clusters of different distances; see Supplementary Figures 1, 2 for an overview of the specific clusters at each sonority distance value). Based on reviewers’ suggestions, we ran additional post-hoc models that take into account that /r/ might occupy a different place on the sonority scale than the one assigned by us. In the first post-hoc model, /tr/ and /kr/ were assigned a sonority distance of 3, in line with a shared position of /r/ and /l/ on the sonority hierarchy between vowels and nasals (cf. Hall, 1992, and explanation in Section 2.1.2) and as depicted in Supplementary Figures 1, 2, while in the second they were assigned a sonority distance value of 1, in line with the realisation of /r/ as the fricative [ʁ]. In the third model, which subdivides the liquids and attests /r/ a higher sonority than /l/ (cf. Hall, 1992), /tr/ and /kr/ were assigned a sonority distance of 4. In the first and third post-hoc model, sonority distance did not show a significant effect. In the second post-hoc model, sonority distance showed a significant effect (*β* = 0.65, *SD* = 0.19, *p* < 0.001), with greater sonority distance leading to higher error rates, i.e., an effect corresponding to that observed for SSP conformity. Moreover, the finer measure of sonority distance does not solve the problem of sibilant and stop ordering on the hierarchy discussed above. It can therefore be abandoned as an alternative to SSP violation as a predictor both on theoretical and on empirical grounds. In their study on recognition of the allophones [kv], [kf], and [kʋ] for German initial <qu> as in Qualm ‘smoke’, Orzechowska and Wiese (2024) reach a different conclusion and suggest that sonority relations determine the ease with which the cluster variants are recognised by German speakers because [kʋ], the variant with the best sonority profile, generally led to the fastest reactions in the lexical decision experiment. However, when looking at their results in detail, an alternative explanation presents itself. While [kv] led to the fastest acceptance of real words, [kʋ] led to the fastest rejection of nonwords. It seems plausible that this is because [kv] is the primary pronunciation variant in German words—(Fuhrhop and Peters, 2013) in spite of it being less well-formed than [kʋ] in terms of sonority distance. We therefore argue that sonority relations might not be the decisive factor in Orzechowska and Wiese (2024), but rather familiarity with different variants, which aligns well with the results of the present study.

As an anonymous reviewer pointed out, it would be informative to see if the patterns of misperception align with other sonority scales in which voiced and voiceless obstruents or /r/ and /l/ are distinguished. As can be seen in the confusion matrix (Supplementary Table 1), the largest proportion of voicing errors occurs on target clusters /tr/ and /kr/ (being reported as /dr/ and /gr/, respectively). Hence, on a sonority scale that distinguishes between voiced and voiceless obstruents, these misperceptions would reduce the sonority distance and still be in conflict with sonority theory. Confusions involving /r/ and /l/ were extremely rare.

#### 2.3.3 Acoustic factors

The effect of onset intensity and the disadvantage for stop–sibilant clusters replicate previous findings (cf. Davidson and Shaw, 2012; Wright, 2001) and are due to differences in noise resistance between the various consonant classes as well as the role of external vs. internal cues to consonant identification. Stops are known to have weak internal cues and rely heavily on external cues, such as formant transitions into the following sound. If that sound is a vowel, then the formant transition is clear and recognition relatively easy. In contrast, if it is a fricative, then it lacks formant structure and recognition of the preceding stop is greatly aggravated. This accounts well for the fact that, over the whole frequency range, stop–sibilant clusters had low recognition rates. Sibilants themselves, on the other hand, have strong internal cues and in the current setting enough energy above the masking noise, which is decisive for successful identification (Moreno-Torres et al., 2017). Hence, clusters with a sibilant in C1 position had low error rates because sibilants themselves have strong internal cues and the following consonant could benefit from its position adjacent to a vowel, which is a good carrier of, for example, stops’ external cues.

One could raise the question whether the frequency effect reported here might be artificially caused by the acoustic effects on consonant classes since the stop-initial clusters with extremely high error rates (/pl, ps, ks/) have medium to low frequencies. We therefore added the binary predictor *stop-initial* to our regression model *post hoc* in order to explore the potential frequency effect independently of the influence of this acoustic disadvantage, but the frequency effect remained robust.^10^ The latter is surprising, considering the picture that emerges from Figure 1. We therefore checked for multicollinearity among the model’s predictors by using the *vif* function in R. All variance inflation factors (VIF) were below 3.20, suggesting no multicollinearity issues. One possible explanation could be that error rates are highest when a cluster is acoustically very similar to another cluster with a much higher frequency, which is the case for the three clusters in question (see Wulfert, 2021, for more detail). Future research should address the perceptual characteristics of these three clusters in more detail.

## 3 Experiment 2: L2 perception

Sonority sequencing did not facilitate the perception of legal L1 sequences in Experiment 1. However, universal principles like the SSP might be more relevant in L2 perception, where the listeners are less familiar with the language-specific distributions. To test this, Experiment 2 employed the same stimuli and task with a group of L2 listeners. We expected that high cluster frequency facilitates perception also in L2 listening, but that listeners might additionally benefit from universally preferred sequences and might also be influenced by the frequencies of the consonant clusters in their L1. The following hypotheses are therefore formulated:

Hypothesis 5: Target-language frequency has a facilitating effect on perception. That means, L2-HF clusters will have lower error rates than L2-LF clusters and at the same time show more false positives.

Hypothesis 6: In addition to L2 frequency, L1 frequency influences perception, such that L1-HF clusters have lower error rates than L1-LF clusters and show more false positives.

Hypothesis 7: SSP-conforming clusters have lower error rates than SSP-violating clusters and perceptual illusions improve the sonority profile of the onset.

Hypothesis 8: The influence of target-language frequencies is greater than that of sonority.

### 3.1 Materials and methods

#### 3.1.1 Participants

Nineteen learners of German living in Sydney (11 female; mean age: 32.53, SD = 12.62) received monetary compensation for their participation. All participants grew up speaking Australian English. One subject reported acquisition of Kannada as his first language but considered English as his primary and more proficient language. All participants reported normal hearing. Their self-reported German levels range from B1 to C2 (B1: n = 2; B2: n = 8; C1: n = 6; C2: n = 3) on the Common European Framework of Reference for Languages (CEFR). All participants gave written informed consent and were free to terminate their participation at any time. Data from three additional participants were excluded from the analyses because they had learned German first as infants.

#### 3.1.2 Material

##### 3.1.2.1 Stimuli

**Figure 5:**
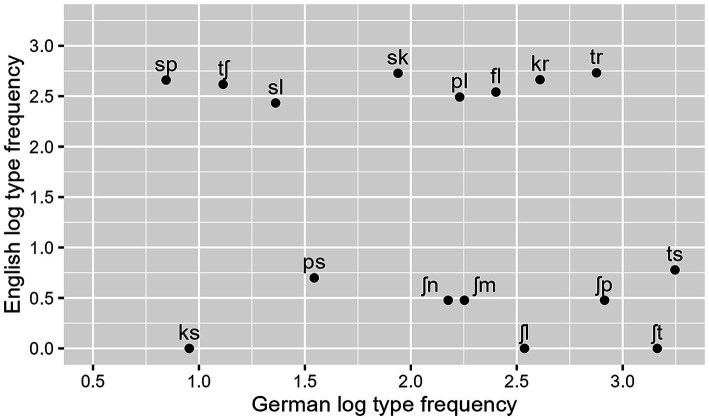
Test clusters’ log type frequencies in English plotted against their log type frequencies in German (both based on WebCELEX).

The stimuli used in Experiment 2 were the same as those used in Experiment 1. Several of the onset clusters used are unattested in English or occur only in a few loanwords. A Pearson correlation shows that the clusters’ English frequencies are not significantly correlated with their German frequencies, *r*(14) = −0.25, *p* = 0.35. Figure 5 gives an overview over the relationship between the clusters’ frequency distributions in German and English.

##### 3.1.2.2 Multi-talker babble

The multi-talker babble files used in the experiment were the same as in Experiment 1. Again, the babble files were randomly assigned to the stimuli for each subject.

#### 3.1.3 Procedure

The main experiment followed the same procedure as in Experiment 1. Participants were tested in a sound-attenuated booth (n = 12) or a quiet library room (n = 7). Before the experiment began, participants were greeted in English unless they started speaking German of their own accord. The experimenter then told them that she would explain the task in German and that they should ask if anything was unclear. Participants were told that they would hear nonsense syllables that sound like German words and received a sheet explaining the spelling system to be used. Unlike in Experiment 1, it explicitly referred to some German spelling conventions (e.g., “ch” wie in “ich,” “‘ch’ as in ‘ich’”; “w” wie in “wer” (entspricht engl. “v”), “‘w’ as in ‘wer’ (corresponds to English ‘v’)”) and explicated the spelling difference between <s-p> = [sp] and <sp> = [ʃp] with reference to English and German pronunciations. Prior to the screening test and the experiment, participants filled in the language background questionnaire and took the German version of the lexTALE test (Lexical Test for Advanced Learners of English, Lemhöfer and Broersma, 2011).

#### 3.1.4 Analysis

Data analyses was the same as in Experiment 1. Since almost half the /sp/−stimuli were transcribed as <sp>, which seems to suggest spelling rather than perception problems, these cases were discarded. Five cases in which the transcription of the onset was not unambiguously interpretable were also excluded, leaving 3,400 of the original 3,520 observations in the data set (exclusion rate: 3.41%).

In addition to the variables that were relevant for the L1 data, English consonant cluster frequencies (see Figure 5) were added to the model to test Hypothesis 6. These were log-transformed type frequencies taken from the English CELEX database (Baayen et al., 1993). Clusters in the test set that are not attested in English (e.g., /ks, ʃl/) were treated as having zero frequency (i.e., assigned a value of 1 prior to log transformation). An interaction between German and English frequencies was also added to the model. In order to specifically test for differences between listener groups, the L1 and L2 data were analysed together in a second logistic regression model with listener group (L1 vs. L2) as a grouping factor. This model included a three-way interaction between German cluster frequencies, English cluster frequencies, and listener group as well as a two-way interaction between SSP violation and listener group.

### 3.2 Results

#### 3.2.1 Accuracy

**Figure 6:**
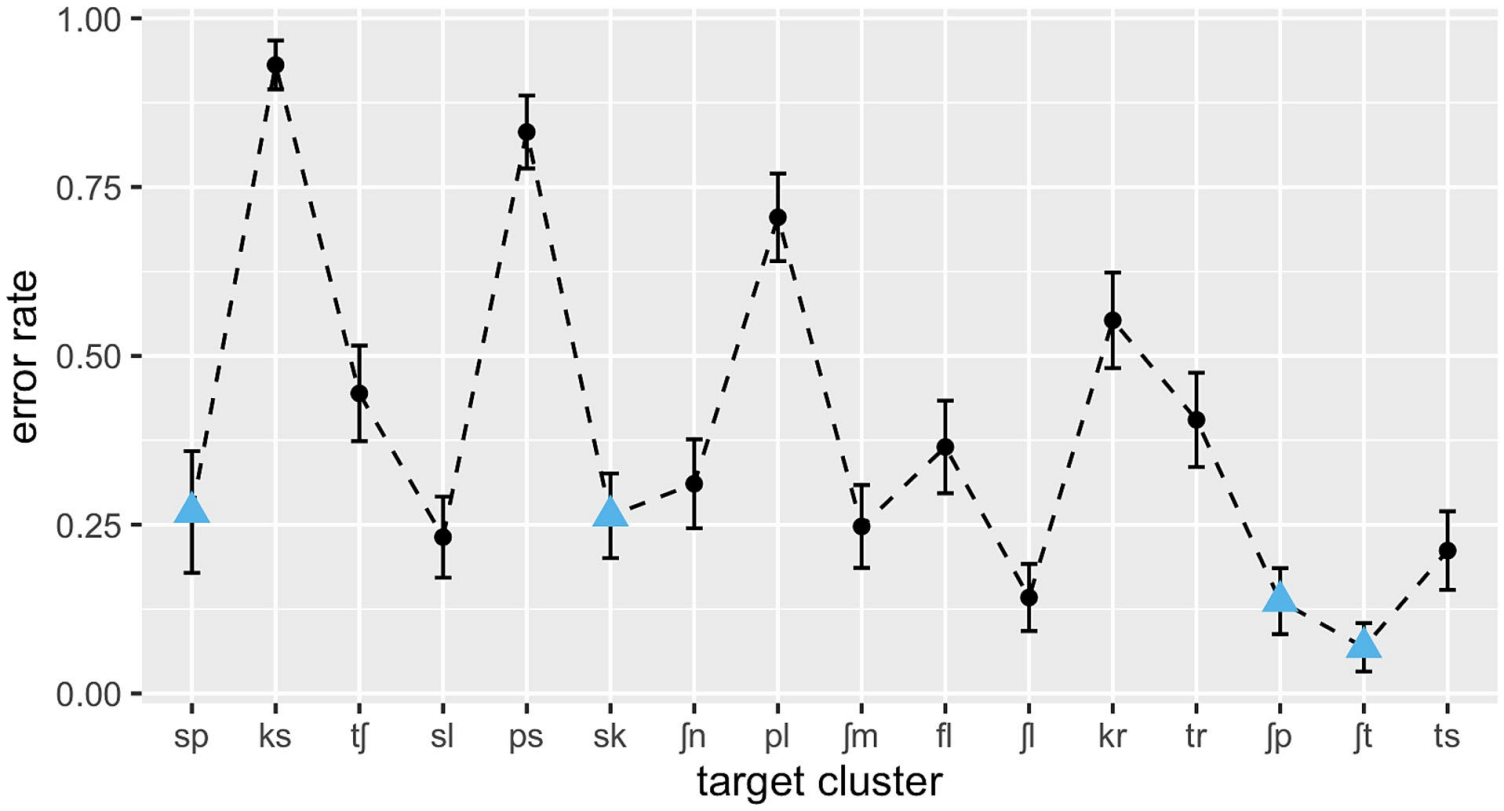
L2 error rates over consonant clusters in ascending order of type frequency; SSP, violating clusters are marked as blue triangles.

The overall error rate in the experiment was 38.6% (SD = 22.3). Error rates ranged from 27 to 59% between participants. There was also considerable variability in error rates over onset clusters, with /ʃt/ showing the lowest error rate (9%). With the exception of /ts/, the stop-initial clusters had the highest error rates; they ranged from 41% for /tr/ to as much as 95% for /ks/. Figure 6 visualises the error rates over consonant clusters.

##### 3.2.1.1 Logistic regression of L2 data

**Figure 7:**
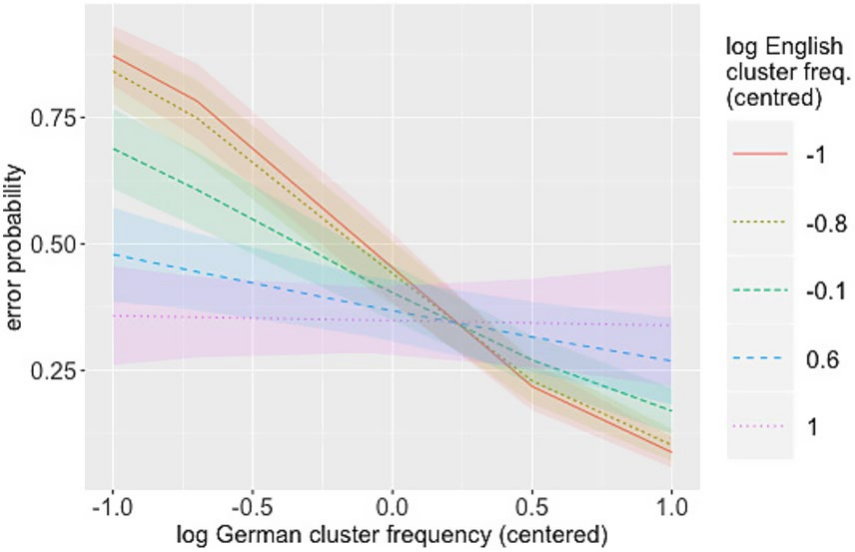
Interaction L2 × L1 frequencies (L2 data).

**Figure 8:**
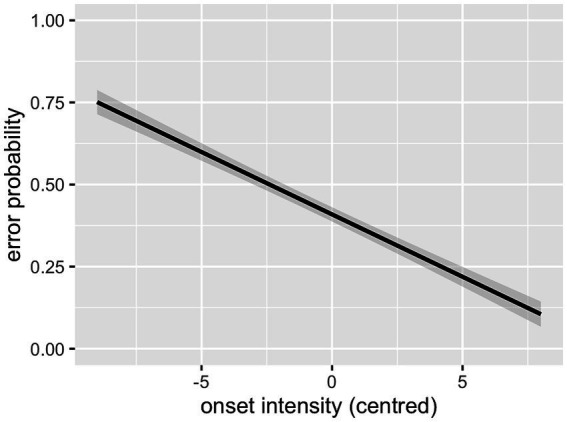
Effect of onset intensity (L2 data).

**Figure 9:**
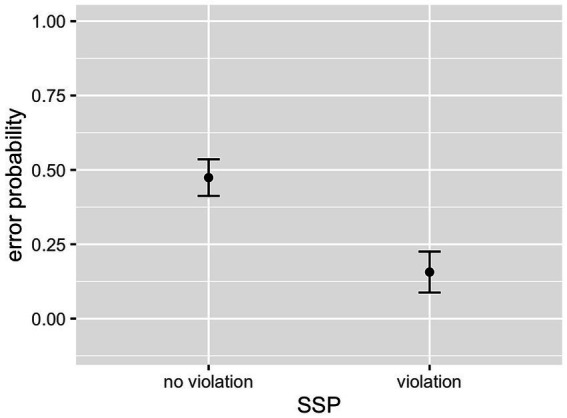
Effect of SSP violation (L2 data).

As can be seen in Table 5, there was a main effect of German log cluster frequency, with higher likelihood of correct identification for higher-frequency clusters. The interaction between German and English log cluster frequencies was significant, revealing a stronger effect of German frequency for clusters with a low frequency in the L1, English (Figure 7). There was no main effect of English log cluster frequencies. Intensity of the onset also showed a main effect, with onsets of higher intensity being recognised with greater accuracy (see Figure 8). As in the Clusters that violate the SSP had a significantly lower error rate than clusters that conform to it (see Figure 9). These findings largely replicate the results of the L1 study: language-specific phonotactics again proved to be relevant to pseudoword perception, while sonority sequencing did not show the predicted facilitating effect. The L2 data also indicate that L1 frequencies are not directly relevant to L2 perception (absence of a main effect of English frequencies) but rather modulate the role of L2 frequencies (interaction between English and German frequencies).

**Table 5:** Summary of the L2 logistic regression model.

Fixed effects				
Effect	β	SE	z	p
(Intercept)	−0.850	0.292	−2.913	0.004
Onset intensity	−0.180	0.043	−4.173	<0.001
German cluster frequency	−1.0879	0.262	−4.155	<0.001
English cluster frequency	−0.208	0.171	−1.213	0.225
SSP violation (no violation)	0.790	0.286	2.768	0.006
German cluster freq × English cluster freq	1.045	0.272	3.835	<0.001

Random effects		
Effect	Variance	SD
Item:target cluster (Intercept)	1.018	1.009
Subject (Intercept)	0.418	0.647
German cluster freq	0.137	0.371
English cluster freq	0.063	0.251
SSP violation	0.098	0.314
German cluster freq × English cluster freq	0.201	0.449
Target cluster (Intercept)	0.328	0.573

Formula: error~ons.intensity + logFreqDE*logFreqEN + SSP.vio + (logFreqDE*logFreqEN + SSP.vio|subjID) + (1|onset.targ/stimulus).

##### 3.2.1.2 Logistic regression of combined L1 and L2 data

There were main effects of German log cluster frequency, SSP violation, and onset intensity (see Table 6). English log cluster frequency did not show an effect. Furthermore, there was a significant main effect of language group: recognition accuracy was higher for the L1 group. Language group interacted with German frequencies: their effect was stronger for the L2 group than for the L1 group. The three-way interaction between German log frequency, English log frequency, and group was also significant, such that English cluster frequencies modulated the effect of German cluster frequencies for the L2 group but not for the L1 group. The interaction between group and sonority violation was not significant. Both groups showed higher error rates for clusters that conform to the SSP than for clusters that violate it.

**Table 6:** Model of L1 and L2 data.

Fixed effects				
Effect	β	SE	z	p
(Intercept)	−1.365	0.266	−5.123	<0.001
German cluster frequency	−0.903	0.257	−3.511	<0.001
English cluster frequency	−0.117	0.167	−0.700	0.484
Group (L1)	−0.430	0.101	−4.271	<0.001
SSP violation (no violation)	0.889	0.281	3.167	0.002
Onset intensity	−0.214	0.039	−5.468	<0.001
German cluster freq × English cluster freq	0.649	0.260	2.490	0.013
German cluster freq × Group	0.164	0.084	1.941	0.052
English cluster freq × Group	0.069	0.049	1.403	0.161
SSP violation × Group	−0.023	0.073	−0.311	0.756
German × English cluster freq × Group	−0.282	0.081	−3.468	<0.001

Random effects		
Effect	Variance	SD
Item:target cluster (Intercept)	0.861	0.928
Group	0.042	0.205
Subject (Intercept)	0.310	0.557
German cluster freq (log)	0.157	0.396
English cluster freq (log)	0.040	0.201
SSP violation	0.072	0.269
German cluster freq × English cluster freq	0.134	0.366
Target cluster (Intercept)	0.414	0.644
Group	0.009	0.094

Formula: error~logFreqDE*logFreqEN*group + SSP.vio*group + ons.intensity + (logFreqDE + SSP.vio|subjID) + (1|onset.targ/stimulus).

#### 3.2.2 Analysis of perceptual repairs

Even among the misperceptions, the vast majority of percepts (97.03%) constituted legal German onsets. A third of the misperceptions were simple onsets, i.e., either deletions of one of the target consonants or insertion of a vowel to break up the target cluster. The most common types of perceptual repair were the same as in the L1 data in most cases. A full confusion matrix can be found in Supplementary Table 2.

##### 3.2.2.1 Frequency comparisons

A comparison of German cluster frequencies of targets and percepts (see Table 7) reveals that about twice as many misperceptions led to a cluster of higher frequency in German than to a cluster of lower frequency, which is in line with Hypothesis 5. In terms of English frequencies, a tendency for perceptual repairs leading to higher-frequency clusters can also be observed (478 vs. 348 cases).

**Table 7:** German frequency comparison of clusters (target vs. cluster reported by L2 listener) in misperceptions.

Frequency comparison	Number of observations
frequency of cluster reported by listener > frequency of target cluster	440 (39.01%)
frequency of cluster reported by listener < frequency of target cluster	223 (19.77%)
frequency of cluster reported by listener = frequency of target cluster	2 (0.18%)
other (listener reported perceiving illegal CC or CCC)	463 (41.05%)

##### 3.2.2.2 Comparison of sonority distances

As regards sonority, it was predicted that sonority distance would increase in misperceptions. However, as Table 8 shows, this happened in only a small minority of cases. In the vast majority of cases, the sonority distance between C1 and C2 either remained the same or was not determinable. The proportion of cases in which the sonority profile of the cluster deteriorated was almost three times as high as the proportion of cases in which it improved.

**Table 8:** Comparison of sonority distances in target clusters vs. clusters reported by listener in the L2 data.

Comparison of sonority distances	Number of observations
sonority distance_reported cluster_ > sonority distance_target_	75 (6.65%)
sonority distance_reported cluster_ < sonority distance_target_	194 (17.20%)
sonority distance_reported cluster_ = sonority distance_target_	451 (39.98%)
sonority distance_reported cluster_ not determinable	408 (36.17%)

### 3.3 Discussion

#### 3.3.1 L2 phonotactics

The analyses showed that L2 perception was strongly influenced by gradient L2 phonotactics. This effect remained robust in the post-hoc models, which were set up to examine whether it was artificially caused by the stop-initial clusters with very high error rates. This supports the hypothesis that target-language phonotactics influences speech perception in L2 listeners as well. In fact, the German frequency effect was even stronger for the L2 listeners than for the L1 listeners and is in line with earlier studies that demonstrated that L2 listeners are able to make use of the structural characteristics of the target language during L2 processing (e.g., Carlson et al., 2016; Hanulíková et al., 2011; Lentz, 2011; Trapman and Kager, 2009; Weber and Cutler, 2006). Expanding on previous studies on categorical effect of L2 phonotactics, our results show that learners acquire gradient L2 phonotactic knowledge. This suggests that phonotactic distributions are acquired separately for each language and are labelled accordingly (cf. Lentz, 2011, p. 164).

The stronger effect in L2 listening may seem surprising at first but can be explained by a skewed distribution of phonotactic sequences in the mental lexicon: the relative frequency difference between the most and least frequent consonant clusters is probably greater for the L2 listeners than for the L1 group. Even with their limited German input, they will have heard the most frequent German phoneme sequences many times, but they may not have heard the least frequent ones at all, which makes infrequent consonant clusters illegal according to their internal representations. As Cutler (2012, pp. 360–361) remarks, “L2 listeners are highly susceptible (to an even greater extent than L1 listeners) to the precise makeup of their vocabulary.” For L2 learners, therefore, the relative frequency difference between the HF clusters and the LF clusters is probably larger, which leads to a more extreme frequency effect. This is in line with the conclusion by Hanulíková et al. (2011, p. 516) that learner frequencies are determined by “[…] a subset of L2 [structures] (most likely the more frequent structures […]).” It casts some doubt on the applicability of the frequency measure used here (i.e., German cluster frequencies based on L1 lexicon entries) for L2 learners. If learners have encountered a skewed phonotactic distribution, a more accurate basis for their mental representations of German sublexical frequencies might be learner corpora (for token frequencies) and learner lexicons (for type frequencies), paralleling the situation in L1 acquisition research in which frequency counts are usually derived from child language corpora.

The L2 listeners’ lack of phonetic knowledge of how to parse the cues for clusters they have never or only rarely encountered (cf. Davidson and Shaw, 2012) probably also contributes to the discrepancy between HF and LF clusters. Without sufficient exposure to appropriate stimuli, listeners cannot acquire knowledge about the relevant phonetic cues for phoneme identification in a specific context, for example identification of initial stops before fricatives, and use cues from their L1 instead. Similar observations have been made by Wilson et al. (2014) for initial stop–nasal clusters.

In addition, L2 listeners might also rely on top-down information to a larger degree than L1 listeners due to their previously acquired knowledge about language-specific phonotactic distributions, which is skewed compared to the actual phonotactic distribution in the language.

#### 3.3.2 L1 phonotactics

However, the influence of L2 phonotactics cannot be discussed without reference to L1 phonotactics. The interaction between German and English frequencies reveals that a strong L2 phonotactic effect emerges only in very low-frequency or non-existent L1 clusters. Similar observations have been made on other linguistic levels, where structures that have equivalents in the learners’ L1 do not show an L2 frequency effect, whereas among those that do not have L1 equivalents, error rates are lower for L2-HF structures than for L2-LF structures (e.g., Fahrner, 2016). This can be interpreted as a transfer effect from the L1: The effect of L2 frequencies (i.e., a learning effect) is greatest for L1-illegal or L1-LF structures because these are the ones which still need to be learned, whereas the listeners are already familiar with the others from their L1. What is perhaps surprising in that respect is the lack of a main effect of English frequencies. If there is an effect of German frequencies on English-illegal clusters but none on English-legal clusters, then it would be expected that their recognition is influenced by their English frequencies instead. It is possible that the L1 frequency effect in the present data was obscured by the high error rates of /pl/ or /kr/, which are frequent in the L1 (see Figure 5) but were probably misperceived due to their phonetic structure (cf. discussion in Section 3.3.1).

In general, the findings are in line with a number of studies reporting effects of L2 (i.e., target-language) phonotactics but not of L1 phonotactics (Boll-Avetisyan, 2011; Lentz and Kager, 2015; Lentz, 2011, ch. 4). How do they relate to previous findings on perceptual illusions in L1-illegal structures? In this experiment, the lack of a main effect of L1 phonotactics attests that not all L1-illegal sequences were repaired. Note, however, that studies reporting perceptual illusions tested structures that are completely illegal in the listeners’ L1. In the present study, only two of the clusters, /ps/ and /ks/, are structurally illegal in the L1 since English does not allow initial heterorganic stop–fricative sequences. All other clusters that do not exist in English, that is, clusters with /ʃ/ in C1 position, have English equivalents with /s/ in C1 position. Likewise, the well-recognised homorganic $/\overset{⌢}{\mathrm{ts}}/$ has the English equivalent $/\overset{⌢}{t\int }/$. Consequently, it is only clusters that are structurally illegal in the L1 that reliably caused perceptual illusions in L2 listeners. They were confused with a single competing cluster more often than they were correctly identified. This might be due to the L2 listeners’ difficulties in interpreting acoustic cues from L2 structures (cf. Davidson and Shaw, 2012; Wilson et al., 2014). This supports the notion of a low-level L1 phonotactic filter through which L2 phonotactic distributions are acquired. It is also in line with feature-based accounts like Albright (2009) and Linzen and O’Donnell (2015), who show that feature-based generalisations are the source of gradient acceptability of phonotactic structures. L1 influence on L2 perception therefore seems to be indirect, while L2 cluster frequencies directly affect their perception.

#### 3.3.3 Sonority sequencing

As in the L1 data, SSP violation of an onset cluster did not yield the expected effect of reduced perceptibility but, on the contrary, led to a perceptual advantage. Clusters that violate the SSP were correctly recognised more often, and the sonority distance between the two consonants of a cluster was increased only in a small minority of misperceptions. Since no phonological theory of sonority would predict this pattern, it is clear that the facilitating effect of SSP-violating clusters must have its origin in a different principle that is correlated with sonority (see Section 2.3.3 above). As the model including both listener groups confirmed, the L2 listeners were no more affected by sonority sequencing than the L1 listeners.^11^

The results of this study contrast with observations in Ulbrich and Wiese (2018), whereby conformity of consonant clusters to the SSP is more important in L2 word learning than L2 phonotactics. The two studies differ not only in terms of the task (identification in noise vs. recollection of word–picture pairs) but also in the composition of test clusters: while all SSP-violating clusters in the present study are legal in German, Ulbrich and Wiese (2018) used a crossed design of L2-legality and SSP-conformity. Therefore, the subjects in the present study may have been more inclined to rely on language-specific phonotactics, which does not serve as reliable guidance in Ulbrich and Wiese’s study. Alternatively, the diverging results could be an indication that sonority takes on a more important role in recollection and learning than in perception. This is very plausible considering that the material to be learned is both new and presented in good listening conditions, thus reducing the influences of language experience and acoustics. Support for the view that sonority sequencing is more important for more conscious tasks than perceptual processing comes from Trapman and Kager’s (2009) study, in which a sonority effect was found in word-likeness ratings but not in lexical decision. They used a broader range of SSP-violating clusters, only one of which contained a sibilant in C1 position. Hence their (null) result in the processing task is less influenced by the good perceptibility of sibilant–stop clusters than the effect found in the present study and thus probably more realistic with respect to the true influence of sonority sequencing on L2 perception.

#### 3.3.4 Acoustic factors

The reduced perceptibility of stops due to poor acoustic cues posed even bigger difficulties for the L2 listeners than for the L1 listeners. In analogy to the L1 data, we also reran our analyses for the L2 data and the dataset including both listeners groups under the inclusion of the binary predictor Stop-initial in a post-hoc fashion (see Supplementary Tables 3, 4). The post-hoc regression revealed that having a stop in C1 position had a detrimental effect on cluster identification, which was only marginally significant when L1 and L2 listener groups were combined. This shows that the L2 listeners suffered more from this acoustic disadvantage. As is to be expected (cf. also Lecumberri et al., 2010), listening in the L2 is harder than in the L1 and this is further aggravated by adverse listening conditions. Furthermore, there was a perceptual advantage for sC clusters in the L2 group, too. Throughout the whole frequency range, these clusters were recognised with an above-average probability. As laid out in Section 2.3.1, this can be best explained by sibilants’ acoustic properties, especially their noise resistance.

## 4 General discussion

This study investigated the identification of initial consonant clusters in noise to determine the extent to which experience with language-specific distributions on the one hand and the SSP as a universal principle on the other hand influence sublexical speech processing in the L1 and the L2. We found that HF clusters are recognised more accurately than LF clusters and are the result of perceptual illusions more often. However, none of the listener groups displayed the expected sonority effect. We will discuss these results in turn.

This frequency result is not unexpected in light of similar effects involving other linguistic units such as words (Grosjean, 1980) and phonemes (Newman et al., 2000), as well as a nonce word production study featuring the same set of onset clusters. It shows that our phonotactic knowledge is gradient and can facilitate sublexical speech perception in a noisy acoustic signal. Hence, the same facilitating effect that Hay et al. (2004) found for heterosyllabic clusters emerged for tautosyllabic clusters in the present experiments.

In addition to frequency of use, acoustic factors proved to be critical for clusters’ perceptibility, as evidenced by the low error rates of sC clusters and particularly high error rates of stop–sibilant clusters. An exception to this pattern is /ts/, which has a very low error rate but is the onset with the highest frequency of use. The difficulty in interpreting especially L2 acoustic cues has been noted before and should also be taken into account in future research.

Results from the two experiments showed that the L1 and the L2 listener group behaved remarkably similar in terms of which clusters were difficult to identify and which were not. In addition, for the Australian group the effect of German cluster frequencies was modulated by their English frequencies. The strong correspondence in the data between the two listener groups shows that, in principle, L2 listeners are susceptible to the same influences as L1 listeners. First of all, they are sensitive to cluster frequencies in the target language. This suggests that they are able to employ distributional knowledge about the target language and are not misled by the frequencies of their L1. The target language frequency effect is even stronger for the L2 group than for the L1 group. This parallels the results of a reading study by Lemhöfer et al. (2011), who found a stronger orthotactic effect for L2 readers than for L1 readers. Similar results have also been obtained for production (repetition accuracy) when comparing children during L1 acquisition to adults (Edwards et al., 2004). In this case, the effect of sublexical transitional probabilities was more extreme for the L1 learners than for the adults. However, the interaction with L1 frequencies in the present data indicates that L1 phonotactics still has an influence on L2 perception, albeit an indirect one.

Moreover, none of the listener groups displayed the expected sonority effect. These results are in stark contrast to those of Berent and collaborators (e.g., Berent et al., 2007; Tamási and Berent, 2015) whose work consistently shows sonority effects in nonword perception and other behavioural tasks and who maintain that sonority sequencing is a relevant principle in language processing and learning. In the present data, a significant effect in the opposite direction was found instead, suggesting that sonority is overturned when in competition with language-specific propensities. This relationship is obscured in Berent’s work which tests consonant clusters that are illegal in the participants’ L1. The sonority effect she finds might to a large degree be driven by other factors that are correlated with it, such as cue robustness in perception, whose influence she also acknowledges (Zhao and Berent, 2016). The present results on perception of sC clusters show that in cases where sonority principles and cue robustness are in conflict, it is clearly perceptual factors that determine perception accuracy and effects in conflict with sonority theory might emerge. In line with previous studies, it is therefore likely that the SSP is a typological generalisation which does not receive much support from phonetic and perceptual empirical evidence (cf. Baroni, 2014; Deschamps et al., 2015; Wilson et al., 2014). It is also worth considering whether the specific sonority scale employed influences the results; whether for example a sonority scale that distinguishes between /l/ and /r/, as considered appropriate for German by Hall (1992), or between voiced and voiceless obstruents would have yielded a sonority effect.

Despite striking similarities in terms of what their perception of consonant clusters is influenced by, there are a number of differences between the two listener groups. Firstly, the tendency for perceptual repair of an uncommon cluster to a common one was more pronounced for the L2 listeners. Especially /ks/ and /ps/ were more frequently reported as /ts/ than they were correctly identified in the L2 group. Likewise, the misperception /sl/ > /ʃl/ is far more common in the L2 group (9.5%) than in the L1 group (0.6%). The absence of /s/ > /ʃ/ confusions in the context of target /ts/ indicates that this is not due to misinterpretation of acoustic cues. Rather, what can be seen here probably demonstrates the impact of expectation based on phonotactic knowledge. In the case of L2 listeners, it probably also involves some sort of hypercorrection: they most likely know that /sl/ is not part of the L1 German repertoire and that English /sl/ corresponds to German /ʃl/ (e.g., *sleep/Schlaf*, *sling/Schlinge*). The L1 listener group was less reluctant to report hearing /sl/. This interpretation may have received more lexical/phonotactic support for the L1 listeners since they know that /sl/ can occur in German speech in words of foreign origin, like *Slang*, *Slalom*, or *slawisch*. For the L2 group, this cluster is probably labelled as belonging to English phonotactics because they are very familiar with it from English but not German words.

Hypercorrection can also be seen in the percentage of illegal percepts: less than 3% of L2 listeners responses contained illegal syllable onsets, whereas for the L1 listeners, the number was more than twice as high.

It must be kept in mind that, in the present study, the two phonotactic systems involved are very similar: structurally, English only differs from German in disallowing initial stop–sibilant and stop–nasal clusters (and allowing consonant–glide clusters). It would be very interesting to compare the present results to data from L2 listeners whose L1 differs more from the target language phonotactically (i.e., is more or less restrictive). In order to further investigate the roles of L1 and L2 gradient phonotactics and their interrelationship in L2 listening, it also seems promising to test for a frequency effect of equivalent L1 clusters in structurally similar languages, for instance, an effect of English /sp/ frequency on German /ʃp/ perception. The low recognition rates for clusters that lack an L1 equivalent could be an indication that L2 structures gain additional support from L1 distributions of equivalent L1 structures. This would contribute greatly to our understanding of how the L1 and L2(s) are organised in bilinguals and how usage changes mental representations.

## Data Availability

The datasets presented in this study can be found in online repositories. The names of the repository/repositories and accession number(s) can be found below: https://osf.io/axd5s.

## Footnotes

^1^Please note that the subdivision of vowels is disregarded in the present study because the different vowels that served as syllable nuclei were balanced across onsets.

^2^Orzechowska and Wiese (2015), as well as related works (e.g., Orzechowska and Dziubalska-Kołaczyk, 2022), list /ʃk/ as a legal onset cluster in German. However, since it does not yield a lexical entry in the German CELEX database and probably would not be accepted as a possible word onset by most German speakers, it is disregarded here. In fact, it seems to be attested only in two place names and two very uncommon loan words (Kleiner et al., 2015).

^3^Please note that this is considered a plateau according to the most common sonority scales (e.g., Clements, 1990). However, even a plateau would be a violation of the SSP. Moreover, for the present purposes, the more fine-grained scale in (1), which separates stops and fricatives by one sonority step, was considered more appropriate.

^4^In natural conversation, /r/ is mostly pronounced as a near-open near-low central vowel (tiefschwa [ɐ]) in coda position. In the stimuli, it was articulated as a consonant (uvular fricative) in order to keep the syllable structure consistent.

^5^Three items (/pliːt/, /slɪt/, /sluːp/) are listed in the English CELEX database. However, we do not see this as a problem for several reasons: Firstly, all of them differed in phonetic detail from their respective English pronunciations; secondly, two of them have a very low frequency of use; and thirdly, the three items together make up less than 2% of the test stimuli.

^6^One stimulus, /ʃpɛf/, was created by splicing two naturally recorded stimuli (/ʃpɛl/ and /spɛf/) because it had been mispronounced in all recordings. Both original recordings were cut at a zero crossing, with the resulting stimulus sounding natural.

^7^ https://librivox.org

^8^ http://audacityteam.org

^9^Note, however, that Baroni (2014) used white noise to mask his stimuli, while multi-talker babble was used in the present study.

^10^For reasons of space, the model will not be reported here, but see the post-hoc model for the L1 and L2 data in Supplementary Table 4, where the factor *stop-initial* is also included.

^11^To rule out the possibility that our measure of SSP violation was too coarse to capture effects of sonority sequencing, we set up a post-hoc model including Sonority Distance (sum-coded) instead of SSP violation, as we did in Experiment 1. Only the level corresponding to a Sonority Distance of −1 turned out to be significantly different from the grand mean (*p* = 0.006), again indicating *lower* error probabilities for these clusters and thus not providing any support for sonority theory.

## Supplementary material

Supplementary Figure 1 “Clusters’ intensity distribution”: Distribution of the stimulus clusters’ intensities; clusters are grouped by sonority distances.

Supplementary Figure 2 “Clusters’ duration distribution”: Distribution of the stimulus clusters’ durations; clusters are grouped by sonority distances.

Supplementary Table 1 “L1 Confusion Matrix”: Rows show target clusters, columns listeners’ responses (order of consonant clusters follows token frequencies); columns C1 and C2 report cases in which only one of the component consonants was perceived as a simple onset, column voice reports voicing errors (e.g., perception of /dr/ for target /tr/) and column other sums up all the remaining confusions; the sum row reports the total of responses (correct and false positives) each stimulus cluster received, with numbers indicating the percentage of responses in relation to the number presentations; note that the value for /sp/ > /ʃp/ confusions is missing because these cases had to be excluded from the analysis; confusions with a single competitor above 20 are printed bold, cells are shaded grey for confusions that are more frequent than correct perception of the target.

Supplementary Table 2 “L2 Confusion Matrix”: Rows show target clusters, columns listeners’ responses (order of consonant clusters follows token frequencies); columns C1 and C2 report cases in which only one of the component consonants was perceived as a simple onset, column voice reports voicing errors in at least one of the consonants, and column other sums up all remaining confusions; numbers represent percentage; note: value for /sp/ > /ʃp/ confusions missing because these cases were excluded from the analysis); confusions with a single competitor above 20 are printed bold, cells are shaded grey for confusions that are more frequent than correct perception of the target.

Supplementary Table 3 “Post-hoc L2 perception logistic regression model”: Formula: error~ons.intensity + logFreqDE * logFreqEN + SSP.vio + stop.initial + (logFreqDE * logFreqEN + SSP.vio|subjID) + (1|onset.targ/stimulus).

Supplementary Table 4 “Post-hoc logistic regression model of L1 and L2 data”: Formula: error~logFreqDE*logFreqEN*group + SSP.vio*group + ons.intensity + stop-initial + (logFreqDE*logFreqEN + SSP.vio|subjID) + (1|onset.targ/stimulus).

Supplementary Table 5 “Post-hoc L2 perception logistic regression model featuring Sonority Distance”: Formula: error~ons.intensity + logFreqDE * logFreqEN + SD + (logFreqDE * logFreqEN + SD|subjID) + (1|onset.targ/stimulus).
