# Supplementary figures and images for: Frequency of use and sonority sequencing in first- and second-language consonant cluster perception: facilitation is language-specific
Source: Front Psychol. 2025 Aug 18;16:1483046. doi: 10.3389/fpsyg.2025.1483046 (PMC12399542; doi:10.3389/fpsyg.2025.1483046)

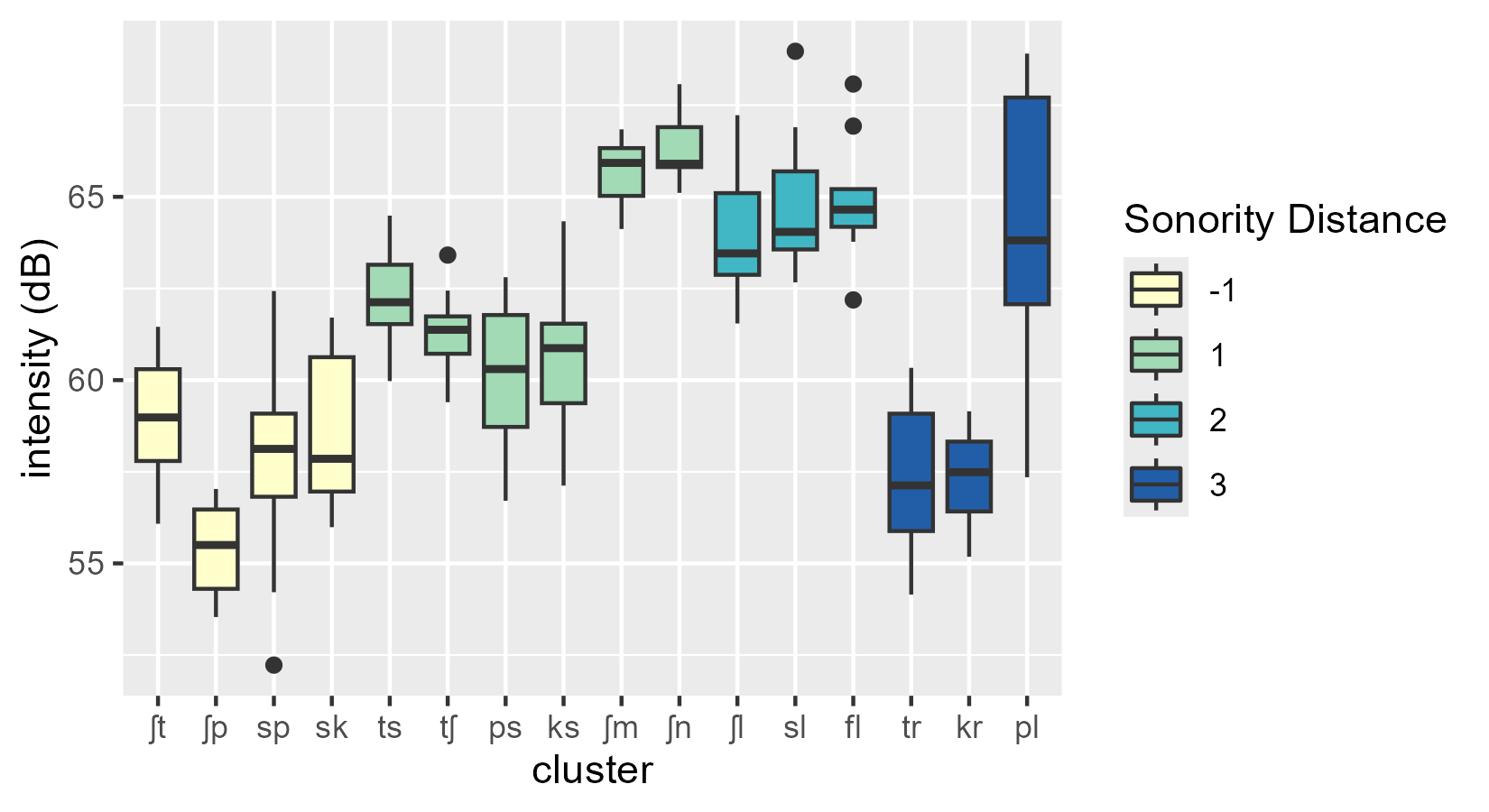

Supplement: Supplementary Figure 1 — “Clusters’ intensity distribution”: Distribution of the stimulus clusters’ intensities; clusters are grouped by sonority distances. [file Image_1.PNG]

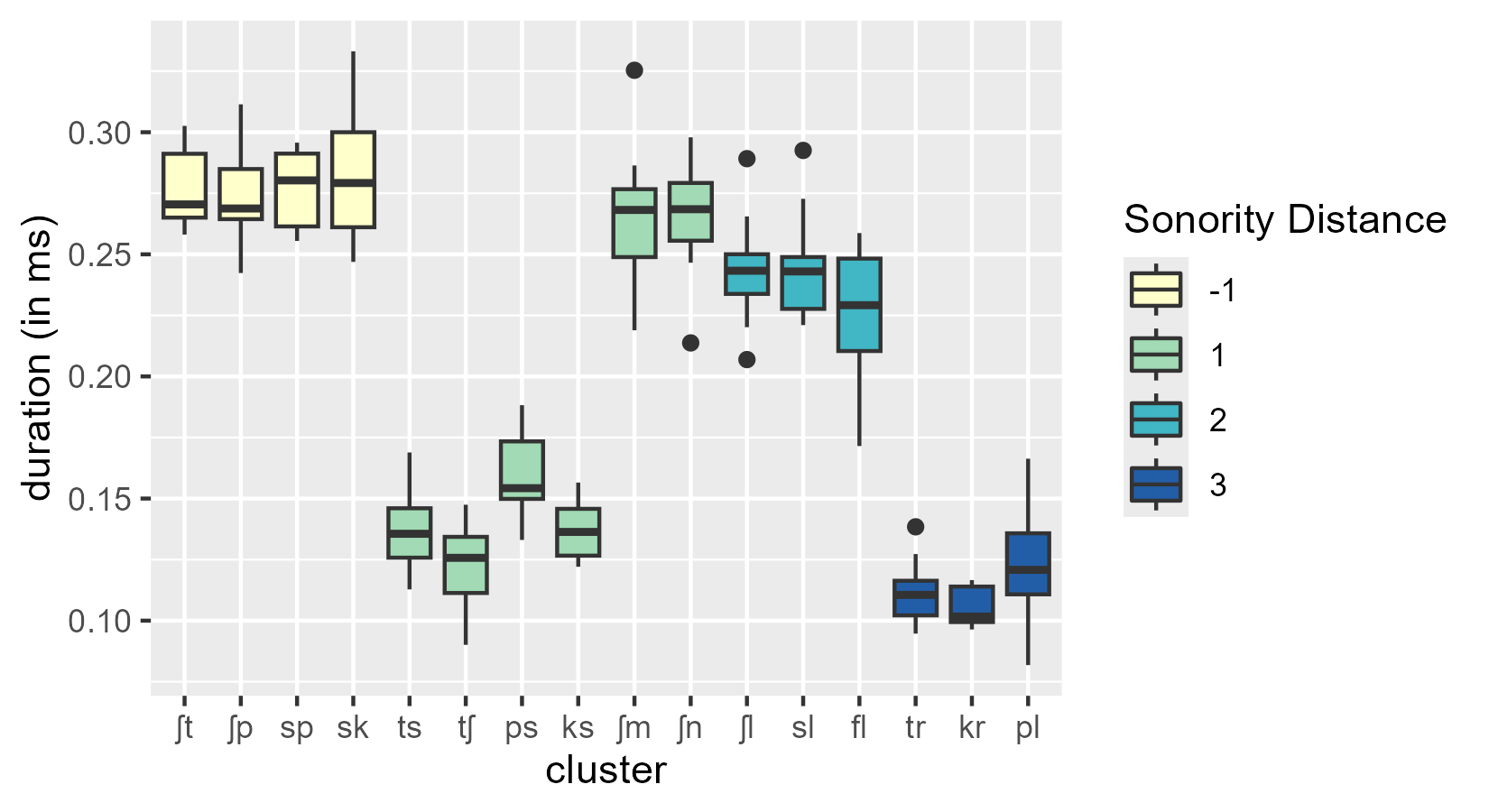

Supplement: Supplementary Figure 2 — “Clusters’ duration distribution”: Distribution of the stimulus clusters’ durations; clusters are grouped by sonority distances. [file Image_2.PNG]
