# Supplementary material for: Frequency of use and sonority sequencing in first- and second-language consonant cluster perception: facilitation is language-specific
Source: Front Psychol. 2025 Aug 18;16:1483046. doi: 10.3389/fpsyg.2025.1483046 (PMC12399542; doi:10.3389/fpsyg.2025.1483046)
Supplement: Supplementary Table 1 — “L1 Confusion Matrix”: Rows show target clusters, columns listeners’ responses (order of consonant clusters follows token frequencies); columns C1 and C2 report cases in which only one of the component consonants was perceived as a simple onset, column voice reports voicing errors (e.g., perception of /dr/ for target /tr/) and column other sums up all the remaining confusions; the sum row reports the total of responses (correct and false positives) each stimulus cluster received, with numbers indicating the percentage of responses in relation to the number presentations; note that the value for /sp/ > /ʃp/ confusions is missing because these cases had to be excluded from the analysis; confusions with a single competitor above 20 are printed bold, cells are shaded grey for confusions that are more frequent than correct perception of the target. [file Table_1.DOCX]

Table 1: Confusion matrix for consonant clusters in L1 perception

Rows show target clusters, columns listeners’ responses (order of consonant clusters follows token frequencies); columns *C1* and *C2* report cases in which only one of the component consonants was perceived as a simple onset, column *voice* reports voicing errors (e.g., perception of /dr/ for target /tr/) and column *other* sums up all the remaining confusions; the sum row reports the total of responses (correct and false positives) each stimulus cluster received, with numbers indicating the percentage of responses in relation to the number presentations; note that the value for /sp/ > /ʃp/ confusions is missing because these cases had to be excluded from the analysis; confusions with a single competitor above 20 are printed bold, cells are shaded grey for confusions that are more frequent than correct perception of the target.

|  | **ts** | **ʃt** | **ʃp** | **tr** | **kr** | **ʃl** | **fl** | **ʃm** | **pl** | **ʃn** | **sk** | **ps** | **sl** | **tʃ** | **ks** | **sp** | **C1** | **C2** | **voice** | **other** |
| --- | --- | --- | --- | --- | --- | --- | --- | --- | --- | --- | --- | --- | --- | --- | --- | --- | --- | --- | --- | --- |
| **ts** | 82 | 0 | 0 | 0 | 0 | 0 | 0 | 0 | 0 | 0 | 0 | 0*.*9 | 0 | 0*.*3 | 1*.*4 | 0*.*3 | 0 | 12 | 0 | 3*.*1 |
| **ʃt** | 0 | 92*.*6 | 4*.*3 | 0 | 0 | 0*.*3 | 0 | 0 | 0 | 0 | 0 | 0 | 0 | 0 | 0 | 0*.*6 | 0 | 0 | 0*.*6 | 1*.*7 |
| **ʃp** | 0 | 1*.*7 | 90 | 0 | 0 | 0 | 0 | 0*.*9 | 0 | 0 | 0*.*3 | 0 | 0 | 0 | 0 | 2*.*9 | 0 | 0 | 0 | 4*.*3 |
| **tr** | 0 | 0*.*3 | 0*.*3 | 72 | 0*.*3 | 0 | 0 | 0 | 0 | 0 | 0 | 0 | 0 | 0 | 0 | 0 | 1*.*1 | 0*.*9 | 19*.*1 | 6 |
| **kr** | 0 | 0 | 0*.*3 | 0*.*3 | 71*.*4 | 0 | 0 | 0 | 0 | 0 | 0 | 0 | 0 | 0 | 0 | 0 | 3*.*1 | 5*.*4 | 12*.*9 | 6*.*6 |
| **ʃl** | 0 | 0*.*3 | 0*.*3 | 0 | 0 | 89*.*4 | 5*.*1 | 0 | 0*.*3 | 0*.*6 | 0 | 0 | 2 | 0 | 0 | 0 | 0 | 0 | 0 | 2 |
| **fl** | 0 | 0 | 0 | 0 | 0 | 3*.*7 | 78*.*3 | 0 | 3*.*7 | 0 | 0 | 0 | 0 | 0 | 0 | 0 | 0*.*9 | 2*.*6 | 0*.*9 | 10 |
| **ʃm** | 0 | 0 | 0*.*3 | 0 | 0 | 0*.*6 | 0*.*3 | 85*.*7 | 0 | 6*.*9 | 0 | 0 | 0 | 0 | 0 | 0 | 0*.*6 | 1*.*7 | 0 | 4 |
| **pl** | 0 | 0 | 0*.*3 | 0 | 0*.*3 | 2 | 8*.*6 | 0 | 36*.*7 | 0 | 0*.*3 | 0 | 0 | 0 | 0 | 0*.*3 | 0*.*9 | 18*.*3 | 6 | 26*.*4 |
| **ʃn** | 0 | 0 | 0*.*3 | 0 | 0 | 4*.*3 | 0*.*3 | 8 | 0 | 82*.*6 | 0 | 0 | 0 | 0 | 0 | 0 | 0*.*9 | 2 | 0 | 1*.*7 |
| **sk** | 0 | 3*.*7 | 6*.*6 | 0 | 0 | 0 | 0 | 0 | 0 | 0 | 78*.*6 | 0 | 0*.*3 | 0 | 0*.*3 | 6*.*3 | 0*.*3 | 0 | 0*.*6 | 3*.*4 |
| **ps** | **28*.*9** | 0 | 2 | 0 | 0 | 0 | 0 | 0 | 0 | 0 | 0 | 33*.*7 | 0 | 0 | 4*.*9 | 2*.*3 | 0 | **22*.*3** | 0*.*3 | 5*.*7 |
| **sl** | 0*.*3 | 0 | 0 | 0 | 0 | 0*.*6 | 2*.*9 | 0 | 0 | 0 | 0*.*6 | 0 | 80*.*9 | 0 | 0 | 0 | 0 | 0 | 0 | 14*.*9 |
| **tʃ** | 1*.*1 | 1*.*4 | 0 | 0 | 0 | 0 | 0 | 0 | 0 | 0 | 0 | 0 | 0 | 71*.*9 | 0*.*3 | 0 | 9*.*5 | 15*.*2 | 0 | 0*.*6 |
| **ks** | **43*.*8** | 0 | 0 | 0 | 0 | 0 | 0 | 0 | 0 | 0 | 1*.*1 | 2 | 0 | 0 | 36*.*4 | 0 | 0 | 10*.*9 | 2*.*3 | 3*.*4 |
| **sp** | 0*.*5 | 1 | *−* | 0 | 0 | 0 | 0 | 0 | 0 | 0 | 13*.*2 | 0 | 0 | 0 | 0 | 72*.*7 | 0 | 1 | 1*.*5 | 10*.*2 |
| sum | 156.6 | 101.0 | 104.7 | 72.3 | 72.0 | 100.9 | 95.5 | 94.6 | 40.7 | 90.1 | 94.1 | 36.6 | 83.2 | 72.2 | 43.3 | 85.4 |  |  |  |  |
