# Supplementary material for: Frequency of use and sonority sequencing in first- and second-language consonant cluster perception: facilitation is language-specific
Source: Front Psychol. 2025 Aug 18;16:1483046. doi: 10.3389/fpsyg.2025.1483046 (PMC12399542; doi:10.3389/fpsyg.2025.1483046)
Supplement: Supplementary Table 2 — “L2 Confusion Matrix”: Rows show target clusters, columns listeners’ responses (order of consonant clusters follows token frequencies); columns C1 and C2 report cases in which only one of the component consonants was perceived as a simple onset, column voice reports voicing errors in at least one of the consonants, and column other sums up all remaining confusions; numbers represent percentage; note: value for /sp/ > /ʃp/ confusions missing because these cases were excluded from the analysis); confusions with a single competitor above 20 are printed bold, cells are shaded grey for confusions that are more frequent than correct perception of the target. [file Table_2.DOCX]

### Table 2: Confusion matrix for consonant clusters in L2 perception

Rows show target clusters, columns listeners’ responses (order of consonant clusters follows token frequencies); columns *C1* and *C2* report cases in which only one of the component consonants was perceived as a simple onset, column *voice* reports voicing errors in at least one of the consonants, and column *other* sums up all remaining confusions; numbers represent percentage; note: value for /sp/ > /ʃp/ confusions missing because these cases were excluded from the analysis); confusions with a single competitor above 20 are printed bold, cells are shaded grey for confusions that are more frequent than correct perception of the target.

|  | **ts** | **ʃt** | **ʃp** | **tr** | **kr** | **ʃl** | **fl** | **ʃm** | **pl** | **ʃn** | **sk** | **ps** | **sl** | **tʃ** | **ks** | **sp** | **C1** | **C2** | **voice** | **other** |
| --- | --- | --- | --- | --- | --- | --- | --- | --- | --- | --- | --- | --- | --- | --- | --- | --- | --- | --- | --- | --- |
| **ts** | 79*.*0 | 0*.*5 | 0 | 0 | 0 | 0 | 0 | 0 | 0 | 0 | 0 | 0 | 0 | 0 | 0 | 0 | 0*.*5 | 16*.*4 | 0 | 3*.*7 |
| **ʃt** | 0 | 91*.*4 | 6*.*8 | 0 | 0 | 0 | 0 | 0 | 0 | 0 | 0 | 0 | 0 | 0 | 0 | 0*.*5 | 0 | 0 | 0 | 1*.*4 |
| **ʃp** | 0 | 1*.*8 | 85*.*9 | 0 | 0 | 0 | 0 | 0*.*5 | 0 | 0 | 0 | 0 | 0 | 0 | 0 | 5*.*0 | 0 | 0 | 0 | 6*.*8 |
| **tr** | 0 | 0 | 0 | 59*.*1 | 0*.*5 | 0 | 0 | 0 | 0 | 0 | 0 | 0 | 0 | 0 | 0 | 0 | 2*.*7 | 0*.*5 | **27*.*3** | 10 |
| **kr** | 0*.*5 | 0*.*5 | 0 | 1*.*8 | 45*.*0 | 0 | 0 | 0 | 0 | 0 | 0 | 0 | 0 | 0 | 0 | 0 | 10*.*9 | 3*.*6 | **24*.*5** | 13*.*2 |
| **ʃl** | 0 | 0*.*5 | 0 | 0 | 0 | 85*.*9 | 2*.*7 | 0 | 0 | 1*.*4 | 0 | 0 | 5*.*0 | 0*.*5 | 0 | 0 | 1*.*4 | 0*.*5 | 0 | 2*.*3 |
| **fl** | 0 | 0 | 0 | 0 | 0 | 6*.*4 | 64*.*8 | 0*.*9 | 3*.*2 | 0 | 0 | 0 | 1*.*4 | 0 | 0 | 0 | 0*.*5 | 5*.*0 | 0*.*9 | 16*.*9 |
| **ʃm** | 0 | 0 | 0*.*5 | 0 | 0 | 0*.*9 | 0 | 75*.*5 | 0 | 3*.*6 | 0 | 0 | 0 | 0 | 0 | 0 | 0 | 5*.*5 | 0 | 14*.*1 |
| **pl** | 0 | 0 | 0 | 0 | 0*.*5 | 1*.*4 | 12*.*7 | 0 | 27*.*7 | 0 | 0 | 0 | 0 | 0 | 0 | 0*.*5 | 0*.*9 | 11*.*8 | 11*.*8 | 32*.*7 |
| **ʃn** | 0 | 0 | 0 | 0 | 0 | 0 | 0*.*5 | 18*.*2 | 0 | 69*.*1 | 0 | 0 | 0 | 0 | 0 | 0 | 1*.*8 | 2*.*3 | 0 | 8*.*2 |
| **sk** | 0*.*5 | 4*.*1 | 9*.*5 | 0 | 0 | 0 | 0 | 0*.*5 | 0 | 0 | 73*.*2 | 0 | 0 | 0 | 0 | 5*.*0 | 0*.*5 | 0*.*9 | 0*.*9 | 5*.*0 |
| **ps** | **42*.*6** | 0*.*5 | 0 | 0 | 0 | 0 | 0 | 0 | 0 | 0 | 0*.*5 | 13*.*9 | 0 | 0 | 0 | 0 | 0 | **32*.*4** | 0 | 10*.*2 |
| **sl** | 0*.*5 | 0 | 0*.*5 | 0 | 0 | 9*.*5 | 4*.*1 | 0 | 0 | 0*.*5 | 0 | 0 | 74*.*1 | 0 | 0 | 0 | 0*.*5 | 0 | 0 | 10*.*5 |
| **tʃ** | 2*.*7 | 0*.*5 | 0 | 5*.*5 | 0 | 0 | 0 | 0*.*5 | 0 | 0 | 0 | 0 | 0 | 55*.*3 | 0 | 0 | 7*.*3 | **22*.*8** | 0 | 5*.*5 |
| **ks** | **59*.*6** | 0*.*5 | 0 | 0 | 0 | 0 | 0 | 0 | 0 | 0 | 0 | 0*.*5 | 0 | 0 | 5*.*0 | 0 | 0 | **27*.*1** | 2*.*8 | 4*.*6 |
| **sp** | 0 | 0*.*9 | *−* | 0 | 0 | 0 | 0 | 0 | 0 | 0 | 7*.*2 | 0 | 0 | 0 | 0 | 70*.*3 | 0*.*9 | 1*.*8 | 0*.*9 | 18*.*0 |
|  |  |  |  |  |  |  |  |  |  |  |  |  |  |  |  |  |  |  |  |  |
| **sum** | 185*.*4 | 101*.*2 | 103*.*2 | 66*.*4 | 46*.*0 | 104*.*1 | 84*.*8 | 96*.*1 | 30*.*9 | 74*.*6 | 80*.*9 | 14*.*4 | 80*.*5 | 55*.*8 | 5*.*0 | 81*.*3 |  |  |  |  |
