# Supplementary material for: Frequency of use and sonority sequencing in first- and second-language consonant cluster perception: facilitation is language-specific
Source: Front Psychol. 2025 Aug 18;16:1483046. doi: 10.3389/fpsyg.2025.1483046 (PMC12399542; doi:10.3389/fpsyg.2025.1483046)
Supplement: Supplementary Table 3 — “Post-hoc L2 perception logistic regression model”: Formula: error~ons.intensity + logFreqDE * logFreqEN + SSP.vio + stop.initial + (logFreqDE * logFreqEN + SSP.vio|subjID) + (1|onset.targ/stimulus). [file Table_3.DOCX]

Table 3
Post-hoc L2 perception logistic regression model; Formula: error~ons.intensity + logFreqDE * logFreqEN + SSP.vio + stop.initial + (logFreqDE * logFreqEN + SSP.vio|subjID) + (1|onset.targ/stimulus)

| **Fixed effects**  **Effect** | **β** | **SE** | **z** | **p** |
| --- | --- | --- | --- | --- |
| (Intercept) | -1.088 | 0.271 | 4.009 | < .001 |
| Onset intensity | -0.113 | 0.053 | -2.124 | .034 |
| German cluster frequency | -1.078 | 0.217 | -4.968 | < .001 |
| English cluster frequency | -0.252 | 0.144 | -1.759 | .079 |
| SSP violation | 0.365 | 0.304 | 1.202 | .230 |
| Stop-initial | 1.028 | 0.406 | 2.535 | .011 |
| German cluster freq × English cluster freq | 0.977 | 0.233 | 4.192 | < .001 |
| **Random effects** |  |  |  |  |
| **Effect** | **Variance** | **SD** |  |  |
| Item:target cluster (Intercept) | 1.049 | 1.024 |  |  |
| Subject (Intercept) | 0.419 | 0.648 |  |  |
| German cluster freq | 0.136 | 0.369 |  |  |
| English cluster freq | 0.063 | 0.250 |  |  |
| SSP violation | 0.096 | 0.310 |  |  |
| German cluster freq × English cluster freq | 0.205 | 0.453 |  |  |
| Target cluster (Intercept) | 0.146 | 0.383 |  |  |
| Marginal *R*^2^ = .349; Conditional *R*^2^ = .585 | | | | |
|  |  |  |  |  |
