# Supplementary material for: Frequency of use and sonority sequencing in first- and second-language consonant cluster perception: facilitation is language-specific
Source: Front Psychol. 2025 Aug 18;16:1483046. doi: 10.3389/fpsyg.2025.1483046 (PMC12399542; doi:10.3389/fpsyg.2025.1483046)
Supplement: Supplementary Table 4 — “Post-hoc logistic regression model of L1 and L2 data”: Formula: error~logFreqDE*logFreqEN*group + SSP.vio*group + ons.intensity + stop-initial + (logFreqDE*logFreqEN + SSP.vio|subjID) + (1|onset.targ/stimulus). [file Table_4.DOCX]

Table 4
Post-hoc logistic regression model of L1 and L2 data; Formula: error~logFreqDE*logFreqEN*group + SSP.vio*group + ons.intensity + stop-initial + (logFreqDE*logFreqEN + SSP.vio|subjID) + (1|onset.targ/stimulus)

| **Fixed effects**  **Effect** | **β** | **SE** | **z** | **p** |
| --- | --- | --- | --- | --- |
| (Intercept) | -1.575 | 0.274 | -5.751 | <.001 |
| German cluster frequency | -0.901 | 0.234 | -3.854 | <.001 |
| English cluster frequency | -0.157 | 0.154 | -1.019 | .308 |
| Group (L1) | -0.434 | 0.101 | -4.306 | <.001 |
| SSP violation (no violation) | 0.628 | 0.301 | 2.086 | .037 |
| Onset intensity | -0.180 | 0.046 | -3.943 | <.001 |
| Stop-initial | 0.754 | 0.425 | 1.776 | .076 |
| German cluster freq × English cluster freq | 0.570 | 0.242 | 2.353 | .019 |
| German cluster freq × Group | 0.167 | 0.084 | 1.968 | .049 |
| English cluster freq × Group | -0.071 | 0.049 | 1.438 | .150 |
| SSP violation × Group | -0.021 | 0.072 | -0.293 | .769 |
| German × English cluster freq × Group | -0.283 | 0.081 | -3.483 | <.001 |
| **Random effects** |  |  |  |  |
| **Effect** | **Variance** | **SD** |  |  |
| Item:target cluster (Intercept) | 0.869 | 0.932 |  |  |
| Group | 0.042 | 0.205 |  |  |
| Subject (Intercept) | 0.310 | 0.557 |  |  |
| German cluster freq | 0.157 | 0.396 |  |  |
| English cluster freq | 0.040 | 0.201 |  |  |
| SSP violation | 0.072 | 0.268 |  |  |
| German cluster freq × English cluster freq | 0.135 | 0.367 |  |  |
| Target cluster (Intercept) | 0.316 | 0.562 |  |  |
| Group | 0.009 | 0.093 |  |  |
| Marginal R2 = .282; Conditional R2 = .534 | | | | |
