# Supplementary material for: Frequency of use and sonority sequencing in first- and second-language consonant cluster perception: facilitation is language-specific
Source: Front Psychol. 2025 Aug 18;16:1483046. doi: 10.3389/fpsyg.2025.1483046 (PMC12399542; doi:10.3389/fpsyg.2025.1483046)
Supplement: Supplementary Table 5 — “Post-hoc L2 perception logistic regression model featuring Sonority Distance”: Formula: error~ons.intensity + logFreqDE * logFreqEN + SD + (logFreqDE * logFreqEN + SD|subjID) + (1|onset.targ/stimulus). [file Table_5.DOCX]

**Language Background Questionnaires**

**L1 group – German original**

1. Alter:

2. Geschlecht: □ weiblich □ männlich

3. Händigkeit: □ RechtshänderIn □ LinkshänderIn

4. Studienfächer (auch frühere):

5. Geburtsort:

6. Sind Sie deutsche/r MuttersprachlerIn? □ nein □ ja

7. Sprechen Sie einen deutschen Dialekt/deutsche Dialekte? □ nein □ ja

Falls ja, welche(n) und wie oft/gut? (siehe Skala)

5 = (fast) täglich

4 = ein- bis mehrmals pro Woche

3 = selten, aber ich beherrsche den Dialekt aktiv

2 = selten (Dialekt ist etwas eingerostet)

1 = ich beherrsche den Dialekt kaum noch

8. Kommen oder kamen Sie regelmäßig mit einem oder mehreren deutschen Dialekten in Berührung (z.B. durch Familie, FreundInnen, PartnerIn)? □ nein □ ja

Falls ja, mit welchem/n? Wie vertraut ist er/sind sie Ihnen?

4 = sehr vertraut

3 = relativ vertraut

2 = nicht so vertraut

1 = kaum vertraut

9. Sprechen Sie weitere Sprachen? □ nein □ ja

Falls ja, welche und wie gut (siehe Skala)?

5 = nahezu muttersprachlich

4 = sehr gut

3 = gut

2 = mittelmäßig

1 = nicht besonders gut

10. Haben oder hatten Sie Hörprobleme? □ nein □ ja und zwar:

11. Liegen bei Ihnen in der Familie Hörschädigungen vor? □ nein □ ja und zwar:

**L1 group – English translation**

1. Age:

2. Gender: □ female □ male

3. Handedness: □ right-handed □ left-handed

4. Fields of study (including past ones):

5. Birth place:

6. Are you a native speaker of German? □ no □ yes

7. Do you speak one or more German dialects? □ no □ yes

If yes, which one(s) and how well? (see scale)

5 = (almost) on a daily basis

4 = once to several times per week

3 = rarely, but I have active command of the dialect

2 = rarely (The dialect has become a bit rusty)

1 = I hardly know the dialect anymore

8. Do you or did you regularly come into contact with one or more German dialects (e.g., through family, friends, partner)? □ no □ yes

If yes, with which dialect(s)? How familiar are you with it/them?

4 = very familiar

3 = relatively familiar

2 = not very familiar

1 = hardly familiar

9. Do you speak other languages? □ no □ yes

If yes, which one(s) and how well (see scale)?

5 = almost native speaker

4 = very well

3 = well

2 = average

1 = not very well

10. Do you or did you have hearing impairments? □ no □ yes, namely:

11. Are there hearing impairments in your family? □ no □ yes, namely:

**L2 group – German original**

1. Alter:

2. Geschlecht: □ weiblich □ männlich □ anderes

3. Studienfächer (auch frühere):

4. Was ist/sind Deine Muttersprache(n)?

5. Seit wie vielen Jahren lernst Du Deutsch?

Seit ___ Jahren

6. Wie alt warst Du, als Du anfingst, Deutsch zu lernen?

___ Jahre alt

7. Wie schätzt Du Deine Deutschkenntnisse ein? (siehe separates Blatt) [official CEFR descriptions were provided on a separate sheet]

8. Warst Du schon einmal in Deutschland/Österreich/der Schweiz?

□ nein □ ja*→* Falls ja, wann und für wie lange?

9. Wie oft übst Du folgende Tätigkeiten auf Deutsch aus? (pro Tag/Woche/Monat/Jahr)

• lesen: Mal pro _____

• hören: Mal pro _____

• schreiben: Mal pro _____

• sprechen: Mal pro _____

10. Kommst oder kamst Du regelmäßig mit einem oder mehreren deutschen Dialekten

in Berührung (z.B. durch FreundInnen, PartnerIn, Medien)? □ nein □ ja

Falls ja, mit welchem/n? Wie vertraut ist er/sind sie Dir?

4 = sehr vertraut

3 = relativ vertraut

2 = nicht so vertraut

1 = kaum vertraut

11. Sprichst Du weitere Sprachen? □ nein □ ja

Falls ja, welche und wie gut (siehe Skala)?

5 = nahezu muttersprachlich

4 = sehr gut

3 = gut

2 = mittelmäßig

1 = nicht besonders gut

12. Hast oder hattest Du Hörprobleme? □ nein □ ja und zwar:

13. Liegen bei Dir in der Familie Hörschädigungen vor? □ nein □ ja und zwar:

**L2 group – English translation**

1. Age:

2. Gender: □ female □ male □ other

3. Fields of study (including past ones):

4. What is/are your native language(s)?

5. For how many years have you been learning German?

For ___ years

6. How old were you when you started learning German?

___ years old

7. How do you estimate your proficiency in German? (see separate sheet) [official CEFR descriptions were provided on a separate sheet]

8. Have you ever been to Germany/Austria/Switzerland?

□ no □ yes*→* If yes, for how long?

9. How often do you do the following activities in German (per day/week/month/year)

• reading: times per _____

• listening: times per _____

• writing: times per _____

• speaking: times per _____

10. Do you or did you regularly come into contact with one or more German dialects (e.g., through friends, partner, the media)? □ nein □ ja

If yes, with which dialect(s)? How familiar are you with it/them?

4 = very familiar

3 = relatively familiar

2 = not very familiar

1 = hardly familiar

11. Do you speak other languages? □ no □ yes

If yes, which one(s) and how well (see scale)?

5 = almost native speaker

4 = very well

3 = well

2 = average

1 = not very well

12. Do you or did you have hearing impairments? □ no □ yes, namely:

13. Are there hearing impairments in your family? □ no □ yes, namely:
